# Efficacy of guided and unguided web‐assisted self‐help for parents of children with attention‐deficit/hyperactivity disorder and oppositional defiant disorder: A three‐arm randomized controlled trial

**DOI:** 10.1111/jcpp.14153

**Published:** 2025-03-10

**Authors:** Manfred Döpfner, Julia Plück, Kerstin Daniela Rosenberger, Marie‐Theres Klemp, Judith Mühlenmeister, Laura Wähnke, Martin Hellmich, Stephanie Schürmann, Christina Dose

**Affiliations:** ^1^ Department of Child and Adolescent Psychiatry, Psychosomatics and Psychotherapy, Faculty of Medicine and University Hospital Cologne University of Cologne Cologne Germany; ^2^ Faculty of Medicine and University Hospital Cologne, School for Child and Adolescent Cognitive Behavior Therapy (AKiP) University of Cologne Cologne Germany; ^3^ Faculty of Medicine and University Hospital Cologne, Institute of Medical Statistics and Computational Biology (IMSB) University of Cologne Cologne Germany

**Keywords:** ADHD, oppositional defiant disorder, school children, parent training, e‐health

## Abstract

**Background:**

Empirical evidence supports the efficacy of behavioral online parent training. However, further large trials in school‐age children with externalizing behavior problems and analyses on the impact of additional therapist support are needed. This three‐arm randomized controlled trial examined the efficacy of guided and unguided web‐assisted self‐help (WASH) for parents of children with externalizing behavior problems.

**Methods:**

Parents of 431 children (6–12 years) with elevated externalizing symptoms were randomly assigned to either treatment as usual (TAU), a 6‐month behavioral WASH intervention (WASH+TAU), or WASH plus telephone‐based support (WASH+S+TAU). Assessments took place at baseline and at 3, 6, and 12 months. The primary outcome was child externalizing symptoms as rated by a clinician blinded to condition; secondary outcomes were parent‐rated child externalizing symptoms, internalizing symptoms, functional impairment, quality of life, parenting practices, and parental internalizing symptoms. (German Clinical Trials Register (DRKS): DRKS00013456; URL: https://drks.de/search/de/trial/DRKS00013456; registered on January 3rd 2018).

**Results:**

Linear mixed models for repeated measures revealed a significant overall intervention effect on blinded clinician‐rated externalizing symptoms at 6 months in both the intention‐to‐treat sample and per‐protocol samples, with at least 25% (PP25) or 40% treatment utilization (PP40), respectively (intention‐to‐treat: *p* = .017). Subsequent pairwise comparisons revealed a greater symptom reduction in WASH+S+TAU than in the other conditions (intention‐to‐treat: WASH+S+TAU vs. WASH+TAU: *p* = .029, *d* = −0.28, 95% CI [−0.54, −0.03]; WASH+S+TAU vs. TAU: *p* = .009, *d* = 0.34 [−0.59, −0.09]). At 12 months, a significant overall effect on blinded clinician‐rated externalizing symptoms only emerged in the PP40 sample (*p* = .035). Secondary analyses revealed an overall effect on child functional impairment at 12 months (intention‐to‐treat and per‐protocol analyses) and on negative parenting behaviors at 6 months in the PP40 sample. For both variables, pairwise comparisons demonstrated significant differences between WASH+S+TAU and TAU.

**Conclusions:**

Parent‐directed WASH is effective in reducing blinded clinician‐rated externalizing symptoms, but only when combined with additional support.

## Introduction

Behavioral parent training has proven effective in the treatment of externalizing behavior disorders such as attention‐deficit/hyperactivity disorder (ADHD) and oppositional defiant disorder (ODD) (Daley et al., 2014; Mingebach, Kamp‐Becker, Christiansen, & Weber, 2018). It is suggested that self‐directed parenting interventions like online parent training overcome frequently reported barriers to accessing face‐to‐face treatment, including limited local service availability, fear of stigmatization, or limited time and financial resources (McGoron & Ondersma, 2015). Meta‐analyses have demonstrated that behavioral online parent training, either alone or in combination with therapist support, has small to moderate effects on child behavior problems and child emotional problems, mostly small effects on parent‐related variables like negative discipline strategies, parenting confidence, parenting satisfaction, and aspects of parental mental health, and large effects on positive parenting behaviors and the quality of parent–child interactions (Spencer, Topham, & King, 2020; Thongseiratch, Leijten, & Melendez‐Torres, 2020). Focusing on studies in children with externalizing behavior problems, a meta‐analysis by Florean, Dobrean, Păsărelu, Georgescu, and Milea (2020) found small effects on child behavior problems, parenting behavior, parent distress, and parenting efficacy. Moreover, online parent training proved to be noninferior to both a self‐directed intervention based on written materials (Florean et al., 2020; Sanders, Dittman, Farruggia, & Keown, 2014) and face‐to‐face parent management training (PMT) (Engelbrektsson et al., 2023; Ghaderi, Kadesjo, Bjornsdotter, & Enebrink, 2018; Prinz, Metzler, Sanders, Rusby, & Cai, 2022).

Regarding the benefit of additional guidance, meta‐analyses conclude that additional therapist support does not enhance the effects of online parent training on child behavior problems (Florean et al., 2020; Spencer et al., 2020). Descriptively, however, the mean effect size for therapist‐led interventions was larger than that for online parent training alone in the meta‐analysis by Florean et al. (2020), which focused on children with behavior problems. Moreover, a study directly comparing online parent training with and without telephone‐based therapist support yielded a significant, moderate group difference in parent‐rated intensity of behavior problems in favor of the support condition (*d* = 0.50; Day & Sanders, 2018).

In sum, despite some promising evidence for the efficacy of behavioral online parent training in children with externalizing behavior disorders, the impact of additional therapist support requires further study. Moreover, most larger studies in clinical samples focused on younger children. For instance, in the meta‐analysis by Florean et al. (2020), which focuses on internet‐based parenting interventions for behavior problems, eight of the 15 included studies focused on children younger than 9 years, with mean ages smaller than 6 years in at least seven studies. Three further studies at least included preschool‐age children. The particular consideration of school‐age children is important as ADHD symptoms change with age and as most guidelines on the treatment of ADHD provide age‐specific treatment recommendations (e.g. Deutsche Gesellschaft für Kinder‐ und Jugendpsychiatrie, Psychosomatik und Psychotherapie [DGKJP] et al., 2018; National Institute for Health and Care Excellence, 2018). It might not be appropriate to extrapolate study results for younger children to older ones, highlighting the need for studies on self‐help interventions in this particular age group.

Thus, the present three‐arm randomized controlled trial (RCT) examined the efficacy of behavioral web‐assisted self‐help (WASH) for parents of children with externalizing behavior problems by comparing a behavioral WASH intervention combined with therapist telephone support as an adjunct to ongoing routine clinical care (WASH+S+TAU), WASH without any additional support (WASH+TAU), and a treatment‐as‐usual condition (TAU) in a large sample of children with elevated ADHD and/or ODD symptoms (6–12 years). We expected that children in the WASH+S+TAU condition would show a greater reduction in clinician‐rated externalizing symptoms (primary outcome) than children in the WASH+TAU condition, and that children in WASH+S+TAU or WASH+TAU would demonstrate a greater decline than children in the TAU condition. Furthermore, we exploratively analyzed the intervention effects on children's comorbid symptoms, functional impairment, and quality of life, and on positive and negative parenting behavior and parental symptoms of depression, anxiety, and stress. Finally, we exploratively examined predictors of the treatment outcome.

## Methods

### Participants and recruitment

Participants were parents of children with externalizing behavior problems, fulfilling the following inclusion criteria (Döpfner et al., 2020): (a) child age between 6;0 and 12;11 years, (b) sufficient German‐language skills of parents to understand the self‐help materials, (c) child (suspected) diagnosis of ADHD by a local healthcare provider, and (d) elevated clinician‐rated ADHD and/or ODD symptoms according to the Diagnostic Checklist for Attention‐Deficit/Hyperactivity Disorder (DCL‐ADHD; Döpfner & Görtz‐Dorten, 2017) and/or the ODD scale of the Diagnostic Checklist for Oppositional Defiant Disorder and Conduct Disorder (DCL‐ODD/CD; Döpfner & Görtz‐Dorten, 2017). These scales were rated by trained members of the study staff based on a parent telephone interview (ILF‐EXTERNAL; Görtz‐Dorten, Thöne, & Döpfner, 2021; Thöne et al., 2020). Symptoms were considered as elevated if a mean subscale score or the total score on one of the aforementioned checklists was over 1.5 standard deviations above the mean score of a representative norm sample on the corresponding scale of a parent‐rated questionnaire comprising the same items (cf. Döpfner & Görtz‐Dorten, 2017). Exclusion criteria were a diagnosis of autism spectrum disorder (ASD), intellectual disability, or the need for inpatient treatment. The assessment of these three exclusion criteria relied on the information provided by the referring local healthcare provider; the presence or absence of these exclusion criteria was not further checked by study staff. Despite the high comorbidity between ADHD and ASD, we decided to exclude children with ASD as the WASH intervention used in the present study was not specifically tailored to this patient group and might not meet the specific needs of this patient group. While the WASH intervention used in this study mainly focuses on the modification of problem‐maintaining factors, the modification of triggering conditions is of particular importance in the treatment of children with ASD. Moreover, parents of children with ASD might have problems to identify themselves with the exemplary descriptions of problem behaviors provided in the WASH program. It is conceivable that the WASH intervention used in the present study is a useful addition to ASD treatment in children with comorbid ADHD. However, due to the remote nature of the intervention, we could not make sure that children with ASD would receive adequate treatment in addition to WASH, which would meet their specific needs. Thus, we decided to exclude children with comorbid ASD and ADHD.

For recruitment, written information about the study was sent to 4,310 registered pediatricians and 730 registered child psychiatrists across Germany between December 2017 and February 2020.

The a priori planned overall sample size was *n* = 495 (Döpfner et al., 2020). Assuming an effect size of *d* = 0.30, based on previous studies using written materials (Dose et al., 2017; Kierfeld, Ise, Hanisch, Goertz‐Dorten, & Doepfner, 2013) and studies on internet‐based interventions (Sourander et al., 2016) as well as two active conditions, an alpha level of 5%, a power of 80%, and a correlation of 0.50 between baseline (T_1_) and postassessment (T_3_) data, the required sample size for a two‐sided analysis of covariance (ANCOVA) was *n* = 132 per group. An additional 25% of participants was planned to be recruited to account for potential dropout and cluster effects. For further details on sample size calculations, see Döpfner et al. (2020).

### Study design

**Figure 1:**
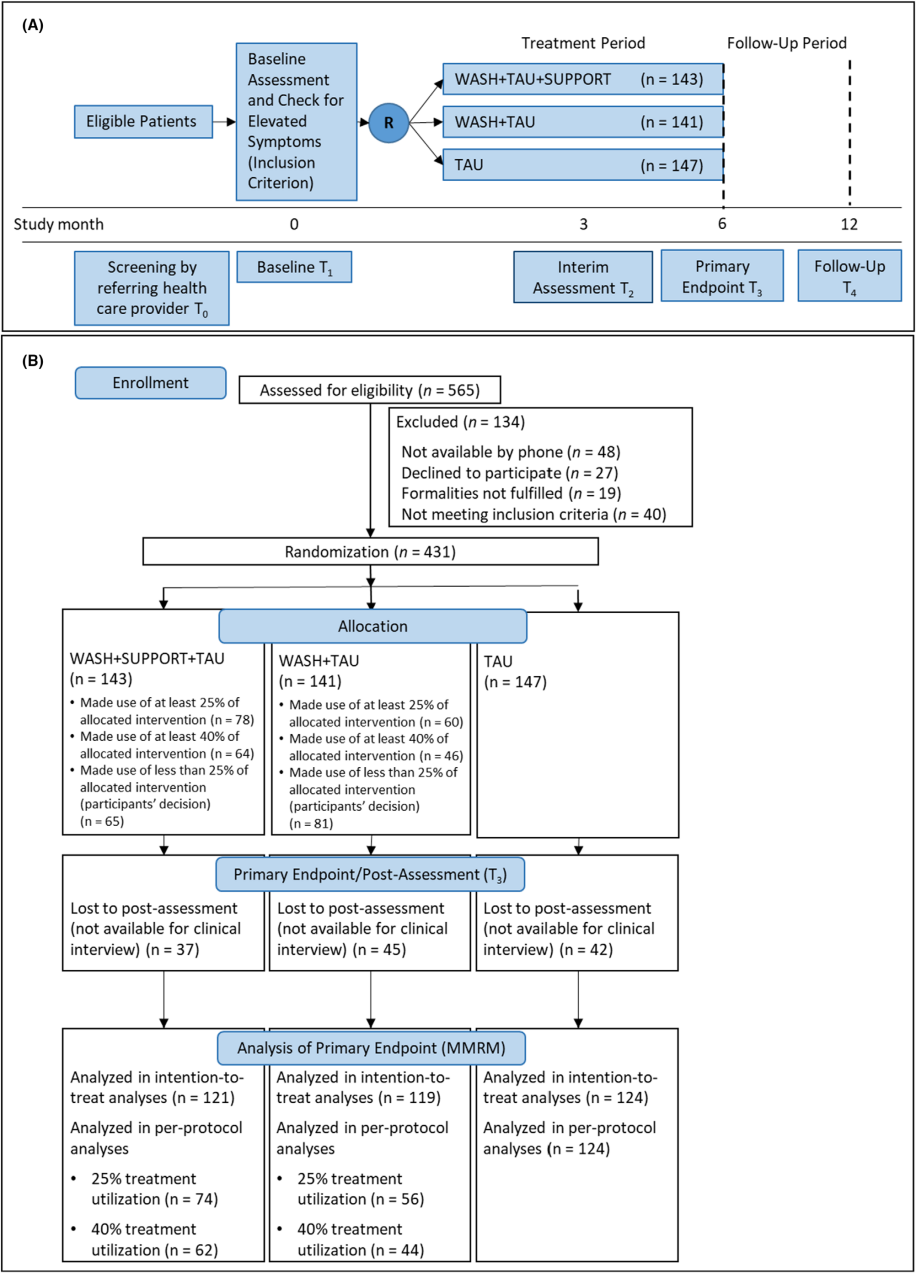
Trial design (A) and participant flow (B)

This RCT used a three‐arm parallel‐group design (see Figure 1A). Participating parents were randomly assigned to one of three treatment conditions: (1) WASH+S+TAU, (2) WASH+TAU, or (3) TAU. There were no restrictions regarding the utilization or change of additional treatments (e.g. pharmacological or psychosocial interventions). Randomization was stratified for child's sex, age, and place of residence (rural vs. urban; differing treatment options). For randomization, the online tool ‘Tenalea’ (ALEA, FormsVision BV, Abcoude, NL) was applied; the allocation ratio was 1:1:1. Assessments took place at baseline (T_1_), 3 months (interim assessment, T_2_), 6 months (post, T_3_), and 12 months (follow‐up, T_4_).

### WASH intervention

The behavioral WASH intervention was based on the ‘Therapy Program for Parents of Children with Hyperkinetic and Oppositional Problem Behavior’ (Döpfner, Schürmann, & Frölich, 2019) and the self‐help book ‘Wackelpeter & Trotzkopf’ (Döpfner & Schürmann, 2017). The program mainly describes techniques of behavior modification and contingency management. It comprises four modules: (1) dealing with problem behaviors (including definition of individual problem situations, psychoeducation about coercive parent–child interactions, definition of family rules, effective methods of communicating demands, and techniques of contingency management), (2) psychoeducation, (3) promoting positive parent–child interactions, and (4) self‐care for parents. Each module is divided into several sub‐modules (see Table S1 and Döpfner et al., 2020).

Parents in both intervention groups received recommendations regarding the sequence of working through the modules; however, all modules were accessible at any time.

Parents randomized to WASH+S+TAU additionally participated in up to six, approximately fortnightly telephone consultations (approx. 20 min) during the first 3 months of the intervention, serving to motivate them to continue with the intervention and to help with the implementation of single techniques. The consultations did not follow a predefined structured protocol but were based on parents' needs. The therapists were pedagogues or psychologists in training for behavioral child and adolescent psychotherapy, who were especially trained for the telephone consultations. That is, they were trained in all study‐specific procedures and received an introduction to the structure and contents of the WASH program and a guideline for the telephone consultations (of note, as outlined above, the consultations were basically based on the parents' needs). All therapists had previously provided telephone consultations in the scope of another study on an assisted, manual‐based self‐help intervention, and had already received training in conducting telephone consultations as part of this other study (though the consultations were more structured in the other study). Thus, they were experienced in this modality of treatment. The consultations were recorded in audio files and supervised by a senior child and adolescent psychotherapist regularly. Measures to improve treatment adherence in both WASH conditions comprised automatic reminder emails from the WASH system, a permanent contact person within the study staff, and a flexible scheduling of study‐related appointments (especially clinical interviews). Moreover, in WASH+S+TAU, a demand‐oriented and flexible scheduling of the telephone consultations was supposed to enhance treatment adherence.

Behavioral manual‐based interventions with similar contents to the WASH intervention have proven effective in combination with telephone‐based support in RCTs (Kierfeld et al., 2013) and observational studies (Kierfeld & Döpfner, 2006; Mokros et al., 2015), with effects remaining stable in the longer term (Döpfner et al., 2021; Ise, Kierfeld, & Döpfner, 2015).

### Outcome measures

Clinician‐rated child externalizing symptoms (primary outcome) were assessed using the DCL‐ADHD (18 items) and the 8‐item ODD scale of the DCL‐ODD/CD (Döpfner & Görtz‐Dorten, 2017) at all assessment points. The clinicians used the ILF‐EXTERNAL to facilitate the ratings. All 26 items were averaged into an overall Externalizing Symptoms score (DCL‐EXT). The preassessment (T_1_) was conducted before randomization, and was thus blinded to the study condition. At postassessment (T_3_), the interviews were recorded for a second, blinded rating_._

Additionally, parents completed several questionnaires online (via LimeSurvey): child ADHD symptoms using the Symptom Checklist for Attention‐Deficit/Hyperactivity Disorder (SCL‐ADHD; Döpfner & Görtz‐Dorten, 2017), child ODD symptoms using the ODD scale of the Symptom Checklist for Disruptive Behavior Disorders (SCL‐DBD; Döpfner & Görtz‐Dorten, 2017), comorbid symptoms using the Child Behavior Checklist (CBCL; Achenbach & Rescorla, 2001; Döpfner et al., 2014), child quality of life using the KIDSCREEN‐10 (KIDSCREEN Group Europe, 2006; Ravens‐Sieberer et al., 2010), parenting behaviors using the Assessment Scale of Positive and Negative Parenting Behaviors (FPNE; Holas et al., 2024; Imort et al., 2014), and parental internalizing symptoms using the Depression Anxiety Stress Scales (DASS; Lovibond & Lovibond, 1995). All outcome measures have satisfactory psychometric properties. Table S2 provides a detailed description of all outcome measures and their psychometric properties.

### Strategies to handle missing data

For 10 participants with missing blinded DCL‐EXT values at T_3_, the corresponding unblinded values were considered for the respective analyses. Further missing values for the DCL‐EXT, the SCL‐ADHD, and the ODD scale of the SCL‐DBD were replaced based on the following procedures: For participants who did not participate in the complete clinical interview or did not provide the complete questionnaires at T_2_, T_3_, and/or T_4_, we tried to obtain ratings for abridged versions of the measures. If ratings for these abridged versions were available, missing DCL‐EXT, SCL‐ADHD, and SCL‐DBD subscale scores were estimated as follows: First, a regression was fitted with the respective subscale score as dependent variable and the short version as predictor, considering all available time points (T_1_–T_4_) of patients with both measurements (clinical interview/questionnaire and respective short version). Based on the regression estimates (intercept and slope), the missing value of the outcome variable at a specific time point was replaced by the predicted value at that time point. Missing total scores were computed based on the imputed subscale scores. Depending on the outcome variable and the time of measurement, between 2 and 11 observations were replaced by this estimator. If participants did not provide at least one complete dataset for either the primary outcome or any of the secondary outcomes at T_3_ or T_4,_ respectively, they were considered as *study dropouts* for the analyses of that assessment time.

### Statistical analyses

The primary analysis of the primary and secondary outcomes was by intention‐to‐treat. The intention‐to‐treat sample comprised all families who had been randomized (*n* = 431). The analysis dataset per outcome variable comprised all participants of the intention‐to‐treat sample for whom the baseline assessment and at least one follow‐up assessment were available. Moreover, we performed per‐protocol analyses in two subsamples to examine effects of intensity of utilization. Besides all families in the TAU condition, the first per‐protocol sample (PP25/2) comprised families from both WASH conditions who had processed at least 25% of the intervention, with families from the WASH+S+TAU condition additionally required to have participated in at least two telephone consultations. The benchmarks for inclusion in the second per‐protocol sample (PP40/3) were 40% processing of the WASH intervention (both WASH conditions) and participation in at least three telephone consultations (WASH+S+TAU).

To analyze the primary and secondary outcomes, we performed analyses of linear mixed models for repeated measures (MMRM analyses) based on the available data, modeling baseline values of the respective outcome, condition, time (assessment point), condition*time, age, and sex as fixed effects (and using an unstructured first‐order variance–covariance matrix over time). In the main analyses, the change from baseline (T_1_) to postassessment (T_3_) was considered as the dependent variable. In a second set of analyses, we used the change from baseline to follow‐up (T_4_) as the dependent variable. The study conditions were compared by contrasting their estimated marginal means. To control for the familywise error rate, we first performed a joint test of significance for each variable at an alpha level of 5% and, if significant, conducted subsequent pairwise comparisons on the same alpha level. We clearly predefined one primary and several secondary outcomes. To retain statistical power regarding the secondary outcomes, we did not adjust for multiple testing. However, readers may easily apply a Bonferroni correction to specific families of hypotheses of interest to guard against type 1 error inflation. Cohen's *d* was considered as a measure of effect size (Cohen, 1988).

As many participants in the present sample made use of pharmacological and nonpharmacological nonstudy treatments, and as about 40% of the participants did not meet the full research diagnostic criteria for any externalizing disorder according to the clinical interview with the parents conducted at baseline (see results section), we performed some sensitivity analyses to get an impression of the influence of these factors on the main analysis of the primary outcome. First, we included fixed effects for the use of pharmacological (yes/no) and nonpharmacological (yes/no) nonstudy treatments as well as diagnostic status (formal diagnosis of an externalizing disorder/no formal diagnosis), condition*diagnostic status, time*diagnostic status, and condition*time*diagnostic status into the model. Pharmacological and non‐pharmacological nonstudy treatments were incorporated by two fixed effects each: (i) a variable with the baseline values and (ii) a time‐dependent variable with the values of the interim and post assessment. Information on nonstudy treatments were assessed along with the outcome measures at all assessment time points. Missing values at T2–T4 were replaced by last‐observation‐carried‐forward (LOCF). Second, we reran this analysis (without the factors relating to diagnostic status) in the subsamples of children with a formal diagnosis of an externalizing disorder and those without a formal diagnosis.

Predictors of the treatment outcome were examined in each study condition using multiple regression analyses, considering clinician‐rated child externalizing symptoms at postassessment (T_3_) as dependent variable. In a first step, several sociodemographic variables and baseline values of the outcome measures were included as putative predictors into the model. In a second step, a more parsimonious model was derived by iteratively dropping the variable with the highest p‐value until only variables with a p‐value ≤0.2 remained. The interpretation of the findings of the prediction analyses were based on this second, final model per study condition.

Most analyses were performed using Stata (versions 17 and 18, StataCorp LLC, College Station, TX, USA), while data management and descriptive information were processed using SPSS Statistics (v27, IBM Corp., Armonk, NY, USA).

## Results

### Participant flow and baseline data

The participant flow is displayed in Figure 1B. In total, 259 pediatricians and 172 child psychiatrists registered 565 families for the study, of which 431 met the inclusion criteria and were randomized (TAU: *n* = 147, WASH+TAU: *n* = 141, WASH+S+TAU: *n* = 143). About a quarter of the participants in WASH+S+TAU (25.9%), 31.9% of the families in WASH+TAU, and 28.6% of the families in TAU did not provide any postassessment data for the primary endpoint (see Figure 1). The mean age of the children was 9.4 years (*SD* = 1.7); 81.4% were boys. According to the structured preassessment interview, 137 children (31.8%) met diagnostic criteria for ADHD according to ICD‐10, 41 children (9.5%) met diagnostic criteria for ODD, and 84 children (19.5%) for both ADHD and ODD. The remaining 169 children (39.2%) did not fulfill diagnostic criteria for any externalizing disorder but showed elevated clinician‐rated externalizing symptoms on the DCL‐ADHD and/or the ODD scale of the DCL‐DBD (see inclusion criteria). Most participating caregivers were female (91.0%), their mean age was 41.4 years (*SD* = 5.8; range: 26–61 years), and most were currently employed (*n* = 354, 82.1%). There were no significant group differences regarding demographic characteristics or baseline values of the outcome variables (see Table S3).

Post hoc power analyses revealed that the sample (*n* = 307 families with valid primary outcome data at postassessment) was sufficiently large to detect an effect of *d* = 0.42, assuming a power of 80% and including an additional 10% of cases to account for cluster effects. As this effect is only slightly larger than that assumed for the sample size calculations, the sample was considered as sufficiently large to perform the planned analyses.

### Treatment as usual

Participants in all conditions made use of a range of nonstudy treatments (referred to as ‘treatment as usual’ in this study; see Table S4). At baseline, 54.81% of the children took medication and 56.7% made use of at least one nonpharmacological treatment (e.g. psychotherapy, ergotherapy/occupational therapy, parent training), with no group difference in the utilization of any treatment; see Table S4). At the interim assessment point (T2) and at postassessment (T3), pharmacological treatment was reported for 63.1% and 65.0% of the children, respectively. At T2 and T3, there was a significant group difference in the utilization of nonpharmacological treatments, with fewer nonpharmacological nonstudy treatments being reported for TAU than for the other two conditions. On the level of single interventions, there was a group difference in the use of parent training and internet‐based self‐help at these assessment points, but not in the use of any other treatment (see Table S4). This finding might be biased; it is conceivable that the parents referred to the WASH program used in this study and not to a nonstudy intervention here, although we may not verify this. When we excluded internet‐based self‐help, the group difference in the use of nonpharmacological treatments dropped to nonsignificance at T2 but remained significant at T3 (see Table S4). At follow‐up (T4), 64.1% of the participants used pharmacological treatment and 63.4% used at least one nonpharmacological treatment, with the group difference for nonpharmacological treatment being significant. On the level of single interventions, there was a significant group difference in the use of psychotherapy (WASH+S+TAU: 36.4%, WASH+TAU: 17.7%, TAU: 26.7%) and internet‐based self‐help (WASH+S+TAU: 20.5%, WASH+TAU: 24.2%, TAU: 2.3%). When internet‐based self‐help was excluded, the group difference in nonpharmacological treatment at T4 dropped to nonsignificance. The rates of families who did not receive any nonstudy treatment were 21.7% at T1, 13.1% at T2, 16.5% at T3, and 14.5% at T4 (no significant group difference; see Table S4).

### Dropout analyses

Regarding the treatment period from baseline (T_1_) to postassessment (T_3_), 98 participants did not provide a complete dataset for any outcome variable and were thus considered as dropouts. Dropout analyses comparing these participants with the 333 participants providing a complete dataset for at least one outcome variable yielded no significant group differences in demographic or clinical characteristics, except for a lower percentage of currently employed persons among the dropouts in the TAU condition (see Table S5).

### Intervention effects on child externalizing behavior

Intention‐to‐treat MMRM analyses on the intervention period from T_1_ to T_3_ yielded a significant overall effect on child externalizing behavior as rated by a blinded clinician. Subsequent pairwise comparisons revealed that children in WASH+S+TAU experienced a greater symptom reduction than children in WASH+TAU and TAU. The corresponding effect sizes were small. Children in WASH+TAU did not significantly differ from children in TAU regarding the reduction of externalizing symptoms (see Table 1; for descriptive statistics, see Table S6). Comparable results emerged in both per‐protocol samples, with effect sizes being slightly larger (PP25/2: WASH+S+TAU vs. TAU: *d* = −0.46, *p* = .002; WASH+S+TAU vs. WASH+TAU: *d* = −0.34, *p* = .053; PP40/3: WASH+S+TAU vs. TAU: *d* = −0.53, *p* = .001; WASH+S+TAU vs. WASH+TAU: *d* = 0.45, *p* = .024; see Tables S8 and S9).

**Table 1:** Group comparisons regarding the mean change from baseline (T_1_) to postassessment (T_3_) based on mixed model repeated measures (MMRM) analyses: intention‐to‐treat sample

Variable	Condition	Marginal means						Total effect	Pairwise comparisons																	
		T2			T3				TAU vs. WASH						TAU vs. WASH+S						WASH vs. WASH+S					
		*M*	(LB;	UB)	*M*	(LB;	UB)	*p*	*n*_TAU_	*n*_WASH_	*d*	(LB;	UB)	*p*_d_	*n*_TAU_	*n*_WASH+S_	*d*	(LB;	UB)	*p*_d_	*n*_WASH_	*n*_WASH+S_	*d*	(LB;	UB)	*p*_d_
DCL‐EXT Total score	TAU	−0.23	−0.29	−0.18	−0.35	−0.42	−0.28	.017	124	119	−0.05	−0.30	0.20	.688	124	121	−0.34	−0.59	−0.09	.009	119	121	−0.28	−0.54	−0.03	.029
	WASH	−0.30	−0.36	−0.24	−0.37	−0.44	−0.30																			
	WASH+S	−0.38	−0.44	−0.32	−0.48	−0.54	−0.41																			
SCL‐ADHD Total score	TAU	−0.17	−0.24	−0.10	−0.27	−0.35	−0.20	.281	122	103	−0.21	−0.47	0.05	.116	122	110	−0.10	−0.36	0.16	.440	103	110	0.11	−0.16	0.38	.438
	WASH	−0.26	−0.34	−0.18	−0.36	−0.44	−0.28																			
	WASH+S	−0.29	−0.37	−0.22	−0.32	−0.40	−0.24																			
SCL‐DBD ODD scale	TAU	−0.07	−0.15	0.01	−0.14	−0.24	−0.04	.099	121	102	−0.17	−0.44	0.09	.198	121	109	−0.28	−0.54	−0.02	.036	102	109	−0.11	−0.38	0.16	.446
	WASH	−0.13	−0.22	−0.04	−0.24	−0.35	−0.13																			
	WASH+S	−0.22	−0.30	−0.14	−0.30	−0.41	−0.19																			
CBCL Internalizing symptoms	TAU	−1.71	−2.59	−0.83	−2.11	−3.07	−1.15	.109	116	100	−0.01	−0.27	0.26	.958	116	106	−0.25	−0.52	0.01	.060	100	106	−0.24	−0.51	0.03	.085
	WASH	−2.42	−3.38	−1.45	−2.15	−3.23	−1.07																			
	WASH+S	−2.47	−3.40	−1.54	−3.49	−4.52	−2.45																			
CBCL Externalizing symptoms	TAU	−2.43	−3.38	−1.47	−2.64	−3.71	−1.57	.090	116	100	−0.18	−0.45	0.08	.179	116	106	−0.29	−0.55	−0.02	.034	100	106	−0.10	−0.37	0.17	.479
	WASH	−2.32	−3.37	−1.28	−3.76	−4.97	−2.55																			
	WASH+S	−2.96	−3.97	−1.95	−4.37	−5.53	−3.21																			
SCL‐ADHD Functional Impairment	TAU	−0.22	−0.31	−0.13	−0.54	−0.60	−0.47	.120	116	99	−0.25	−0.52	0.02	.070	116	106	−0.22	−0.48	0.04	.101	99	106	0.03	−0.24	0.30	.831
	WASH	−0.29	−0.39	−0.19	−0.63	−0.71	−0.55																			
	WASH+S	−0.30	−0.40	−0.20	−0.62	−0.69	−0.55																			
KIDSCREEN Total score	TAU	0.68	−0.14	1.50	0.72	−0.19	1.62	.953	116	100	0.02	−0.25	0.28	.906	116	106	0.04	−0.22	0.30	.759	100	106	0.02	−0.25	0.30	.861
	WASH	−0.03	−0.93	0.88	0.80	−0.23	1.83																			
	WASH+S	0.87	0.00	1.73	0.93	−0.06	1.91																			
FPNE Positive parenting	TAU	−0.02	−0.06	0.02	0.01	−0.04	0.06	.698	116	100	−0.01	−0.28	0.25	.924	116	106	0.09	−0.17	0.36	.482	100	106	0.11	−0.17	0.38	.451
	WASH	0.03	−0.02	0.07	0.01	−0.05	0.06																			
	WASH+S	0.04	0.00	0.09	0.03	−0.02	0.08																			
FPNE Negative parenting	TAU	−0.03	−0.07	0.01	−0.07	−0.12	−0.03	.136	116	100	−0.02	−0.29	0.24	.860	116	106	−0.25	−0.51	0.02	.066	100	106	−0.22	−0.49	0.06	.119
	WASH	−0.08	−0.12	−0.04	−0.08	−0.13	−0.03																			
	WASH+S	−0.13	−0.17	−0.09	−0.13	−0.18	−0.09																			
DASS Total score	TAU	−0.05	−0.11	0.01	−0.09	−0.16	−0.02	.166	116	100	0.15	−0.12	0.42	.271	116	106	−0.12	−0.38	0.15	.387	100	106	−0.26	−0.53	0.01	.062
	WASH	−0.04	−0.11	0.02	−0.03	−0.11	0.05																			
	WASH+S	−0.12	−0.18	−0.06	−0.14	−0.21	−0.06																			

CBCL, Child Behavior Checklist; *d*, Cohen's *d* (effect size); DASS, Depression Anxiety Stress Scales; DCL‐EXT, Diagnostic Checklist for Externalizing Behavior Disorders; KIDSCREEN, questionnaire to assess health‐related quality of life; LB, lower bound; *M*, mean; *n*, sample size; ODD, Oppositional Defiant Disorder; *p*, significance value; FPNE, Assessment Scale of Positive and Negative Parenting Behavior; SCL‐ADHD, Symptom Checklist for Attention‐Deficit/Hyperactivity Disorder; SCL‐DBD, Symptom Checklist for Disruptive Behavior Disorders; T1, baseline; T2, interim assessment; T3, postassessment; TAU, treatment as usual; UB, upper bound; WASH, web‐assisted self‐help; WASH+S, web‐assisted self‐help and additional support via telephone.

When considering the intervention period from T_1_ to T_4_, an overall significant effect on the reduction of externalizing symptoms only emerged in the PP40/3 sample (see Tables S7–S9). Subsequent pairwise comparisons yielded a small group difference between WASH+S+TAU and TAU (*d* = −0.39, *p* = .014).

In the sensitivity analysis controlling for the use of pharmacological and nonpharmacological interventions and including terms on the children's diagnostic status, the overall effect on blinded clinician‐rated child externalizing symptoms remained significant (*p* = .018). The 2‐ and 3‐way interactions between study condition and diagnostic status as well as study condition, diagnostic status and time were not significant in this model (*p*‐values of the interaction terms ranged between 0.412 and 0.944). As in the main analysis, subsequent pairwise comparisons yielded a significant difference between WASH+S+TAU and WASH+TAU and between WASH+S+TAU and TAU, but not between WASH+TAU and TAU (see Table S10). When the analysis was performed in the subsample of children without a formal diagnosis of an externalizing disorder, the overall effect slightly missed significance (*p* = .060). Likewise, the overall effect was nonsignificant in the subsample of children with a formal diagnosis (*p* = .232; see Table S10).

No overall effect on parent‐rated externalizing symptoms (i.e. on the SCL‐ADHD or the ODD scale of the SCL‐DBD) emerged in any of the analyses (see Table 1 and Tables S7–S9).

### Intervention effects on comorbid symptoms, functional impairment, and quality of life

The MMRM analyses did not yield any intervention effects on parent‐rated child internalizing or externalizing symptoms (CBCL) or child quality of life (KIDSCREEN‐10). However, children in WASH+S+TAU showed a greater reduction in ADHD‐related impairment (SCL‐ADHD Functional Impairment scale) from T_1_ to T_4_ than children in TAU in the intention‐to‐treat sample and in both per‐protocol samples (small effects). In the intention‐to‐treat sample, the pairwise comparisons yielded an additional small significant group difference between TAU and WASH+TAU in favor of WASH+TAU (see Table 1 and Tables S7–S9).

### Intervention effects on parenting behaviors and parental internalizing symptoms

A significant overall effect on negative parenting behavior emerged in the PP40/3 sample at T_3_ but not at T_4_ (see Table 1 and Tables S7–S9). Pairwise comparisons in this sample revealed a significant, small group difference between WASH+S+TAU and TAU (*d* = −0.42, *p* = .009). The group difference between WASH+S+TAU and WASH+TAU narrowly missed significance (*d* = −0.40, *p* = .055; see Table S9).

Regarding positive parenting behavior, we did not detect any immediate effects. However, MMRM analyses in both per‐protocol samples revealed significant effects in the longer term, with subsequent pairwise comparisons demonstrating significant, moderate group differences between WASH+S+TAU and TAU in favor of the latter (PP25/2: *d* = −0.50, *p* = .001; PP40/3: *d* = −0.60, *p* < .001). The difference between WASH+TAU and WASH+S+TAU narrowly missed significance in these two samples (see Tables S8 and S9).

The analyses did not reveal any effects on parental symptoms of depression, anxiety, and stress (see Table 1 and Tables S7–S9).

### Predictors of clinician‐rated child externalizing symptoms at post‐assessment

Clinician‐rated child externalizing symptoms (DCL‐EXT total score) at baseline significantly predicted clinician‐rated child externalizing symptoms at postassessment in all three conditions, with more severe baseline symptoms being predictive of more severe symptoms at postassessment. Parent‐rated overall child emotional and behavioral problems (CBCL total score) at baseline were additionally found to significantly predict treatment outcome in TAU, with more severe emotional and behavioral symptoms being associated with more severe externalizing symptoms at T_3_. Finally, child age and the percentage of processed content of the WASH program were additional significant predictors of treatment outcome in WASH+S+TAU. Here, a younger age and a higher percentage of processed content were associated with less severe externalizing symptoms at postassessment.

### Treatment acceptance and utilization

The majority of participants (*n* = 239, 84.2%) in WASH+TAU and WASH+S+TAU logged in at least once until T_2_, with a binomial test indicating a significantly higher percentage in WASH+S+TAU (88.1%) than in WASH+TAU (80.1%; *p* = .008). The overall number of logins was also higher in WASH+S+TAU (*M* = 5.82, *SD* = 4.43) than in WASH+TAU (*M* = 3.92, *SD* = 4.22; *t*(282) = 3.730, *p* < .001). Eighty‐eight participants (61.5%) in WASH+S+TAU utilized all six telephone consultations, 85.3% utilized at least two, and 81.8% at least three. The mean number of telephone consultations was 4.62 (*SD* = 2.09; range 0–6). On average, the consultations lasted 25.82 min (*SD* = 5.34; range 7.50–52.17).

## Discussion

This three‐arm RCT examined the efficacy of a behavioral WASH intervention for parents of children with externalizing behavior problems in a sample of 6–12‐year‐old children with elevated ADHD and/or ODD symptoms. MMRM analyses and subsequent pairwise comparisons indicated that WASH+S+TAU was superior to both TAU and WASH+TAU in reducing blinded clinician‐rated externalizing symptoms (primary outcome) immediately after the intervention. This result was found in both intention‐to‐treat analyses and per‐protocol analyses in subsamples with at least 25% or 40% treatment utilization, respectively. However, neither of the analyses yielded a group difference in blinded clinician‐rated externalizing symptoms between WASH+TAU and TAU. Effect sizes were small in the intention‐to‐treat analyses and increased in the subsamples with more intense treatment utilization. At 3‐month follow‐up, only the difference between WASH+S+TAU and TAU in the PP40/3 sample remained significant. These results support the efficacy of the WASH intervention combined with additional telephone support, whereas the intervention without additional support does not seem superior to routine clinical care. That is, additional support seems necessary to achieve effects. The vanishing of most of the immediate effects during the follow‐up period might hint at the importance of booster sessions (although this requires further research).

Of note, the use of nonstudy treatments was rather high in all study conditions, and there was a higher use of nonpharmacological interventions in both WASH conditions compared to the TAU condition at T2 and T3 (although this latter finding might be biased, as at least part of parents indicating the use of internet‐based self‐help might refer to the intervention used in this study and not to nonstudy treatment; see results section). In sensitivity analyses controlling for the use of pharmacological and nonpharmacological nonstudy treatments, the overall effect remained significant, and pairwise comparisons pointed to the superiority of WASH+S+TAU over both WASH+TAU and TAU. However, the high use of nonstudy treatments in all conditions generally makes it difficult for the WASH interventions to demonstrate superiority over TAU. A similar problem was observed in the Multimodal Treatment of Attention‐Deficit/Hyperactivity Disorder (MTA) study, which could not demonstrate superiority of behavioral treatment over a community care condition, which received rather intensive treatment (e.g. 68% of the children in the community care condition were treated with medication; Jensen et al., 2001).

Regarding the secondary outcomes, effects emerged for the following variables: (a) negative parenting behavior immediately after the intervention (PP40/3 sample; less negative parenting behavior in WASH+S+TAU than in TAU), (b) positive parenting behavior at 3‐month follow‐up (both per‐protocol samples; but: more positive parenting behavior in TAU than in WASH+S+TAU), and (c) functional impairment at 3‐month follow‐up (intention‐to‐treat and both per‐protocol samples; less impairment in WASH+S+TAU than in TAU). All significant effects were small to moderate at most. No intervention effects were found regarding comorbid symptoms, quality of life, and parental internalizing symptoms.

The present findings correspond to previous studies on behavioral online parent training (cf. Florean et al., 2020) by pointing at small to moderate effects of telephone‐supported online parent training on externalizing symptoms, and provide – albeit limited – support for intervention effects on functional impairment and negative parenting practices. Notably, intervention effects in previous studies were mostly found in parent ratings of behavior problems, with clinical ratings only seldom considered (cf. Florean et al., 2020; Spencer et al., 2020; Thongseiratch et al., 2020). By contrast, the present study only revealed effects on blinded clinical ratings of externalizing symptoms and not on parent ratings. This is also contrary to studies on behavioral face‐to‐face parent training and self‐directed interventions in general (either web‐ or manual‐based), which mostly found effects in parent‐rated outcome measures but not in blinded assessments or clinical observations (Daley et al., 2014; Sonuga‐Barke et al., 2013; Tarver, Daley, Lockwood, & Sayal, 2014). Therefore, and given the divergent results for the blinded‐rated and parent‐rated symptom measures in our study, we cannot completely exclude potential bias associated with the blinded ratings or clinical interviews, for example due to socially desirable responding to an interviewer.

Moreover, previous meta‐analytic comparisons suggest that additional support might not enhance the effects of behavioral online parent training (Florean et al., 2020; Spencer et al., 2020), while our findings clearly suggest the need for additional therapist support to achieve improvements. A previous three‐arm RCT found effects of both supported and merely self‐directed online parent training compared to a waitlist control condition, and additionally revealed the superiority of the condition with additional support in reducing the intensity of behavior problems (Day & Sanders, 2018).

In this study, the differing findings for the WASH conditions with and without additional support may be regarded in the light of utilization: While most participants in both intervention conditions logged in at least once, supporting the acceptance of the intervention, the number of logins was higher in the support condition. Thus, additional support (e.g. via telephone) seems to enhance participants' acceptance or motivation, which might be a prerequisite for symptom improvements. On the other hand, about 12% of the participants in WASH+S+TAU and 20% in WASH+TAU never made use of the program. The fact that these participants enrolled, but did not participate in the program, might be seen in the light of the referral from a local health care provider and the relatively uncomplicated intake procedures. Due to the easy access to the study and the WASH program and the recommendation by their health care provider, some families might have enrolled although their motivation to actually participate and put effort into the intervention was rather low. Also, the initial uptake rate seems to be higher in case of additional support, at least on a descriptive level.

Overall, the discrepant findings between our study and previous studies regarding the role of supported behavioral online parent training suggests the need for further research. For instance, it may be useful to examine specific characteristics of the support offered along with the different online programs to gain a clearer impression of how additional support should look and the level of intensity required to derive additional benefits.

In our own research group, we have conducted studies on behavioral telephone‐assisted self‐help interventions based on written materials, which mostly demonstrated somewhat larger effect sizes for the reduction of externalizing symptoms than the current study (e.g. Dose et al., 2017; Kierfeld et al., 2013). A possible explanation may lie in the low structure of both the use of the online program and the telephone sessions in the present study, with participants navigating through the WASH program according to their interests and needs. Accordingly, the supporting telephone sessions were based on patients' needs, without following a predefined protocol. The WASH intervention and telephone sessions might be improved by providing more structure, guiding parents through the program more intensively, and making single components of the intervention only accessible if others have been processed. The supporting telephone sessions could be adapted accordingly. Indeed, previous research on online parent training, which provided more structure then the intervention used here, yielded somewhat larger effects (Enebrink, Högström, Forster, & Ghaderi, 2012). In this regard, future research could also investigate the specific components of online parent training which determine its efficacy, and their presentation.

The fact that diagnostic status (children with vs. without formal diagnosis) did not demonstrate any significant interactions with study condition and time in the sensitivity analysis indicates that the effect on blinded clinician‐rated child externalizing symptoms does not differ between children with and without a formal diagnosis of an externalizing disorder. However, the overall effect on blinded clinician‐rated child externalizing symptoms approached significance in the subsample of children without a formal diagnosis, but not in the subsample of children without a formal diagnosis. Then again, the power of these latter analyses is limited due to the reduced sample sizes. Given the inconsistent results of the analyses on the influence of the diagnostic status and the limited validity of this variable, we are not able to draw firm conclusions about whether the WASH intervention is more effective in children not meeting diagnostic criteria of an externalizing disorder than in children with a formal diagnosis. There are slight indications that the program is more effective in children with externalizing symptoms below the diagnostic threshold, but this has to be examined further in future studies.

The finding that child age was predictive of clinician‐rated child externalizing symptoms as postassessment in WASH+S+TAU suggests that this kind of intervention might be more effective in younger children (within the considered age group of 6‐ to 12‐year‐olds). Adaptations might be necessary to better suit the needs of children approaching adolescence. For instance, older children could be more involved in interventions like the negotiation of rules, and techniques of problem‐solving and communication training could be integrated, as has been recommended for the treatment of adolescents (Wolraich et al., 2019).

The present findings should be considered in light of several strengths and limitations. Clear strengths concern the large sample size and the comparison of three arms, including a direct comparison of the behavioral WASH intervention with and without additional support. A first limitation concerns the above‐mentioned low structure of the intervention, which might have limited its efficacy. Second, as outlined above, the different findings for different raters of externalizing symptoms impede a clear impression of the effects of the program. Third, as we excluded children with comorbid ASD and ADHD, the generalizability of the findings to children with this frequent comorbidity is limited. The WASH program could be modified to meet the specific needs of other clinical populations, for instance, children with this comorbidity. Fifth, as outlined above, the use of nonstudy, ‘treatment as usual’ interventions was rather high in all study conditions, including TAU. The study was conducted in Germany; the generalizability of the findings to other countries with other supply structures might be limited. Finally, although the WASH intervention was designed to ease treatment access, about 15% of the participants did not use the intervention at all. This rate is lower than in similar studies on both self‐directed interventions and face‐to‐face interventions. However, barriers to the implementation of online parent training should be explored, possibly also using qualitative methods.

## Conclusions

The study supports the efficacy of therapist‐guided behavioral WASH for parents of children with externalizing behavior problems in reducing externalizing behavior symptoms as rated by a blinded clinician based on a parent interview, both immediately after the intervention and at 6‐month follow‐up. Additionally, effects on functional impairment and negative parenting behaviors were found. The WASH intervention without additional support was not superior to routine clinical care, highlighting that this support seems necessary to achieve improvements. As the intervention effects on externalizing symptoms were not replicated in parent‐rated questionnaires, the results should be interpreted with some caution. Moreover, as the effects were smaller than those previously found for more structured telephone‐assisted self‐help interventions based on written materials, future research could focus on a modified, more structured version of the WASH intervention. In the longer term, the parent‐directed WASH intervention could be used to facilitate treatment access and thus make parent training available for a larger group of patients.

## Ethical considerations

The RCT was approved by the Medical Ethical Committee of the University Hospital of Cologne, Germany. All procedures performed in this study involving human participants were in accordance with the ethical standards of the institutional and/or national research committee and with the 1964 Helsinki declaration and its later amendments or comparable ethical standards. All participating parents provided written informed consent for participation.

## Trial registration

The study was registered at the German Clinical Trials Registry (identifier: DRKS00013456, URL: https://drks.de/search/de/trial/DRKS00013456; registered on January 3 2018). The study protocol has been published previously (Döpfner et al., 2020).

**Key points**

- Online parent training has proven effective in children with externalizing disorders. However, larger RCTs in school‐age children are lacking, and the impact of additional therapist support remains unclear.
- This three‐arm RCT examined the efficacy of parent‐directed, web‐assisted self‐help (WASH) in children with elevated externalizing symptoms (6–12 years), comparing three conditions: treatment as usual (TAU), WASH+TAU, and WASH plus telephone‐based support (WASH+S+TAU).
- WASH+S+TAU was superior to both WASH+TAU and TAU in reducing child externalizing symptoms (blinded clinician rating, primary outcome) at postassessment and 6‐month follow‐up. Moreover, the analyses revealed effects on parent‐rated functional impairment and negative parenting behaviors.
- WASH+TAU seemed not superior to TAU, indicating the importance of additional support.
- WASH could facilitate treatment access and enhance the availability of parent training.

## Supporting information


**Table S1** Components of the web‐assisted self‐help intervention.
**Table S2**. Description and psychometric properties of the outcome measures.
**Table S3**. Sociodemographic and clinical characteristics at baseline (intention‐to‐treat sample).
**Table S4**. Nonstudy treatments/treatment as usual.
**Table S5**. Dropout analysis comparing families with and without available postassessment data (T3).
**Table S6**. Descriptive statistics for the primary and secondary outcome measures (based on available data).
**Table S7**. Group comparisons regarding the mean change from baseline (T1) to follow‐up (T4) based on mixed model repeated measures (MMRM) analyses: intention‐to‐treat sample.
**Table S8**. Group comparisons regarding the mean change from baseline (T1) to postassessment (T3) and from baseline to follow‐up (T4) based on mixed model repeated measures (MMRM) analyses: per‐protocol sample (WASH: made use of at least 25% of the WASH intervention; WASH+S: additionally participated in at least 2 telephone consultations).
**Table S9**. Group comparisons regarding the mean change from baseline (T1) to postassessment (T3) and from baseline to follow‐up (T4) based on mixed model repeated measures (MMRM) analyses: per‐protocol sample (WASH: made use of at least 40% of the WASH intervention; WASH+S: additionally participated in at least 3 telephone consultations).
**Table S10**. Group comparisons regarding the mean DCL‐EXT change from baseline (T1) to postassessment (T3) based on mixed model repeated measures (MMRM) analyses: sensitivity analyses in the intention‐to‐treat sample.
**Table S11**. Prediction of blinded clinician‐rated child externalizing symptoms (primary outcome) at T3 in the TAU condition: intention‐to‐treat sample.
**Table S12**. Prediction of blinded clinician‐rated child externalizing symptoms (primary outcome) at T3 in the WASH+TAU condition: intention‐to‐treat sample.
**Table S13**. Prediction of blinded clinician‐rated child externalizing symptoms (primary outcome) at T3 in the WASH+S+TAU condition: intention‐to‐treat sample.

## Data Availability

The dataset can be obtained from the corresponding author upon request.

## References

[jcpp14153-bib-0001] Achenbach, T.M. , & Rescorla, L.A. (2001). Manual for the ASEBA school‐age forms and profiles. Burlington, VT: University of Vermont, Research Center for Children, Youth and Families.

[jcpp14153-bib-0002] Cohen, J. (1988). Statistical power analysis for the behavioral sciences. Hillsdale, London: Lawrence Erlbaum Associates.

[jcpp14153-bib-0003] Daley, D. , Van der Oord, S. , Ferrin, M. , Danckaerts, M. , Doepfner, M. , Cortese, S. , & Sonuga‐Barke, E. (2014). Behavioral interventions in attention‐deficit/hyperactivity disorder: A meta‐analysis of randomized controlled trials across multiple outcome domains. Journal of the American Academy of Child and Adolescent Psychiatry, 53, 835–847. 25062591 10.1016/j.jaac.2014.05.013

[jcpp14153-bib-0004] Day, J.J. , & Sanders, M.R. (2018). Do parents benefit from help when completing a self‐guided parenting program online? A randomized controlled trial comparing Triple P Online with and without telephone support. Behavior Therapy, 49, 1020–1038. 30316482 10.1016/j.beth.2018.03.002

[jcpp14153-bib-0005] Deutsche Gesellschaft für Kinder‐ und Jugendpsychiatrie, Psychosomatik und Psychotherapie (DGKJP) , Deutsche Gesellschaft für Psychiatrie und Psychotherapie, Psychosomatik und Nervenheilkunde , & Deutsche Gesellschaft für Sozialpädiatrie und Jugendmedizin . (2018). Long version of the interdisciplinary evidence‐ and consensus‐based (S3) guideline “Attention‐Deficit/Hyperactivity Disorder (ADHD) in children, adolescents and adults”. AWMF Registration No. 028‐045. Available from https://www.awmf.org/uploads/tx_szleitlinien/028‐045k_S3_ADHS_2018‐06.pdf

[jcpp14153-bib-0006] Döpfner, M. , & Görtz‐Dorten, A. (2017). Diagnostik‐System für Psychische Störungen nach ICD‐10 und DSM‐5 für Kinder und Jugendliche – III [Diagnostic system for mental disorders in childhood and adolescence according to ICD‐10 and DSM‐5]. Göttingen, Germany: Hogrefe.

[jcpp14153-bib-0007] Döpfner, M. , Liebermann‐Jordanidis, H. , Kinnen, C. , Hallberg, N. , Mokros, L. , Benien, N. , … & Dose, C. (2021). Long‐term effectiveness of guided self‐help for parents of children with ADHD in routine care – An observational study. Journal of Attention Disorders, 25, 265–274. 30449268 10.1177/1087054718810797

[jcpp14153-bib-0008] Döpfner, M. , Plück, J. , Kinnen, C. , & Arbeitsgruppe Deutsche Child Behavior Checklist . (2014). Elternfragebogen über das Verhalten von Kindern und Jugendlichen (CBCL/6‐18R). Deutschsprachige Fassung der Child Behavior Checklist for Ages 6–18 von Thomas M. Achenbach [German version of the Child Behavior Checklist for Ages 6–18 by Thomas M. Achenbach]. Göttingen, Germany: Hogrefe.

[jcpp14153-bib-0009] Döpfner, M. , & Schürmann, S. (2017). Wackelpeter und Trotzkopf. Hilfen für Eltern bei ADHS‐Symptomen, hyperkinetischem und oppositionellem Verhalten [Help for parents of children with ADHD symptoms, hyperkinetic and oppositional problem behaviors] (5th edn). Weinheim, Germany: Beltz.

[jcpp14153-bib-0010] Döpfner, M. , Schürmann, S. , & Frölich, J. (2019). Therapieprogramm für kinder mit hyperkinetischem und oppositionellem Problemverhalten [Therapy program for parents of children with hyperkinetic and oppositional problem behavior] (6th edn). Weinheim, Germany: Beltz. 9459672

[jcpp14153-bib-0011] Döpfner, M. , Wähnke, L. , Klemp, M.‐T. , Mühlenmeister, J. , Schürmann, S. , Hellmich, M. , & Plück, J. (2020). Efficacy of web‐assisted self‐help for parents of children with ADHD (WASH) – A three arm randomized trial under field/routine care conditions in Germany. BMC Psychiatry, 20, 76–84. 32085706 10.1186/s12888-020-2481-0 PMC7035664

[jcpp14153-bib-0012] Dose, C. , Hautmann, C. , Buerger, M. , Schuermann, S. , Woitecki, K. , & Doepfner, M. (2017). Telephone‐assisted self‐help for parents of children with attention‐deficit/hyperactivity disorder who have residual functional impairment despite methylphenidate treatment: A randomized controlled trial. Journal of Child Psychology and Psychiatry, 58, 682–690. 27878809 10.1111/jcpp.12661

[jcpp14153-bib-0013] Enebrink, P. , Högström, J. , Forster, M. , & Ghaderi, A. (2012). Internet‐based parent management training: A randomized controlled study. Behavior Research and Therapy, 50, 240–249. 10.1016/j.brat.2012.01.006 22398153

[jcpp14153-bib-0014] Engelbrektsson, J. , Salomonsson, S. , Högström, J. , Sorjonen, K. , Sundell, K. , & Forster, M. (2023). Parent training via internet or in group for disruptive behaviors: A randomized clinical noninferiority trial. Journal of the American Academy of Child & Adolescent Psychiatry, 62, 987–997. 36863414 10.1016/j.jaac.2023.01.019

[jcpp14153-bib-0015] Florean, I.S. , Dobrean, A. , Păsărelu, C.R. , Georgescu, R.D. , & Milea, I. (2020). The efficacy of internet‐based parenting programs for children and adolescents with behavior problems: A meta‐analysis of randomized clinical trials. Clinical Child and Family Psychology Review, 23, 510–528. 32897527 10.1007/s10567-020-00326-0

[jcpp14153-bib-0016] Ghaderi, A. , Kadesjo, C. , Bjornsdotter, A. , & Enebrink, P. (2018). Randomized effectiveness trial of the family check‐up versus internet‐delivered parent training (iComet) for families of children with conduct problems. Scientific Reports, 8, 11486. 30065246 10.1038/s41598-018-29550-z PMC6068169

[jcpp14153-bib-0017] Görtz‐Dorten, A. , Thöne, A.K. , & Döpfner, M. (2021). Interviewleitfäden zum Diagnostik‐System für psychische Störungen für Kinder‐ und Jugendliche (DISYPS‐III‐ILF) [Clinical interview for the diagnostic system for mental disorders in childhood and adolescence]. Bern, Switzerland: Hogrefe.

[jcpp14153-bib-0039] Holas, V. , Thöne, A.K. , Dose, C. , Gebauer, S. , Hautmann, C. , Görtz‐Dorten, A. , … & Döpfner, M. (2024). Psychometric properties of the parent‐rated assessment scale of positive and negative parenting behavior (FPNE) in a German sample of school‐aged children. Child and Adolescent Psychiatry and Mental Health, 18, 1–15. 39681854 10.1186/s13034-024-00850-9 PMC11648292

[jcpp14153-bib-0018] Imort, S. , Hautmann, C. , Greimel, L. , Katzmann, J. , Pinior, J. , Scholz, K. , … & Döpfner, M. (2014). Fragebogen zum positiven und negativen Erziehungsverhalten [Positive and negative parenting questionnaire]. Poster presented at the 32nd Conference for Clinical Psychology and Psychotherapy of the German Society for Psychology, Braunschweig, Germany.

[jcpp14153-bib-0019] Ise, E. , Kierfeld, F. , & Döpfner, M. (2015). One‐year follow‐up of guided self‐help for parents of preschool children with externalizing behavior. Journal of Primary Prevention, 36, 33–40. 25331981 10.1007/s10935-014-0374-z

[jcpp14153-bib-0020] Jensen, P.S. , Hinshaw, S.P. , Swanson, J.M. , Greenhill, L.L. , Conners, C.K. , Arnold, L.E. , & Wigal, T. (2001). Findings from the NIMH multimodal treatment study of ADHD (MTA): Implications and applications for primary care providers. Journal of Developmental & Behavioral Pediatrics, 22, 60–73. 11265923 10.1097/00004703-200102000-00008

[jcpp14153-bib-0021] KIDSCREEN Group Europe . (2006). The KIDSCREEN questionnaires: Quality of life questionnaires for children and adolescents. Lengerich, Germany: Pabst.

[jcpp14153-bib-0022] Kierfeld, F. , & Döpfner, M. (2006). Bibliotherapie als Behandlungsmöglichkeit bei Kindern mit externalen Verhaltensstörungen [Bibliotherapy as a self‐help program for parents of children with externalizing problem behavior]. Zeitschrift für Kinder‐ und Jugendpsychiatrie und Psychotherapie, 34, 377–386. 16981158 10.1024/1422-4917.34.5.377

[jcpp14153-bib-0023] Kierfeld, F. , Ise, E. , Hanisch, C. , Goertz‐Dorten, A. , & Doepfner, M. (2013). Effectiveness of telephone‐assisted parent‐administered behavioural family intervention for preschool children with externalizing problem behavior: A randomised controlled trial. European Child & Adolescent Psychiatry, 22, 553–565. 23463180 10.1007/s00787-013-0397-7

[jcpp14153-bib-0024] Lovibond, S.H. , & Lovibond, P.F. (1995). Manual for the depression anxiety stress scales (DASS). In Psychology foundation monograph (2nd edn). Sydney, Australia: Psychology Foundation of Australia.

[jcpp14153-bib-0025] McGoron, L. , & Ondersma, S.J. (2015). Reviewing the need for technological and other expansions of evidence‐based parent training for young children. Children and Youth Services Review, 59, 71–83. 10.1016/j.childyouth.2017.12.004 PMC5931387 29731531

[jcpp14153-bib-0026] Mingebach, T. , Kamp‐Becker, I. , Christiansen, H. , & Weber, L. (2018). Meta‐meta‐analysis on the effectiveness of parent‐based interventions for the treatment of child externalizing behavior problems. PLoS One, 13, e0202855. 30256794 10.1371/journal.pone.0202855 PMC6157840

[jcpp14153-bib-0027] Mokros, L. , Benien, N. , Mütsch, A. , Kinnen, C. , Schürmann, S. , Wolf Metternich‐Kaizman, T. , & Döpfner, M. (2015). Angeleitete Selbsthilfe für Eltern von Kindern mit Aufmerksamkeitsdefizit‐/Hyperaktivitätsstörung: Konzept, Inanspruchnahme und Effekte eines bundesweiten Angebotes – Eine Beobachtungsstudie [Guided self‐help interventions for parents of children with ADHD – concept, referral and effectiveness in a nationwide trial. An observational study]. Zeitschrift für Kinder‐ und Jugendpsychiatrie und Psychotherapie, 43, 275–288. 26118815 10.1024/1422-4917/a000348

[jcpp14153-bib-0028] National Institute for Health and Care Excellence . (2018). *Attention deficit hyperactivity disorder: Diagnosis and management*. Report No.: NG87. 29634174

[jcpp14153-bib-0029] Prinz, R.J. , Metzler, C.W. , Sanders, M.R. , Rusby, J.C. , & Cai, C. (2022). Online‐delivered parenting intervention for young children with disruptive behavior problems: A noninferiority trial focused on child and parent outcomes. Journal of Child Psychology and Psychiatry, 63, 199–209. 33829499 10.1111/jcpp.13426 PMC9912029

[jcpp14153-bib-0030] Ravens‐Sieberer, U. , Erhart, M. , Rajmil, L. , Herdman, M. , Auquier, P. , Bruil, J. , … & European KIDSCREEN Group . (2010). Reliability, construct and criterion validity of the KIDSCREEN‐10 score: A short measure for children and adolescents' well‐being and health‐related quality of life. Quality of Life Research, 19, 1487–1500. 20668950 10.1007/s11136-010-9706-5 PMC2977059

[jcpp14153-bib-0031] Sanders, M.R. , Dittman, C.K. , Farruggia, S.P. , & Keown, L.J. (2014). A comparison of online versus workbook delivery of a self‐help positive parenting program. The Journal of Primary Prevention, 35, 125–133. 24500106 10.1007/s10935-014-0339-2

[jcpp14153-bib-0032] Sonuga‐Barke, E.J. , Brandeis, D. , Cortese, S. , Daley, D. , Ferrin, M. , Holtmann, M. , & European ADHD Guidelines Group . (2013). Nonpharmacological interventions for ADHD: Systematic review and meta‐analyses of randomized controlled trials of dietary and psychological treatments. American Journal of Psychiatry, 170, 275–289. 23360949 10.1176/appi.ajp.2012.12070991

[jcpp14153-bib-0033] Sourander, A. , McGrath, P.J. , Ristkari, T. , Cunningham, C. , Huttunen, J. , Lingley‐Pottie, P. , … & Unruh, A. (2016). Internet‐assisted parent training intervention for disruptive behavior in 4‐year‐old children: A randomized clinical trial. JAMA Psychiatry, 73, 378–387. 26913614 10.1001/jamapsychiatry.2015.3411

[jcpp14153-bib-0034] Spencer, C.M. , Topham, G.L. , & King, E.L. (2020). Do online parenting programs create change? A meta‐analysis. Journal of Family Psychology, 34, 364–374. 31697102 10.1037/fam0000605

[jcpp14153-bib-0035] Tarver, J. , Daley, D. , Lockwood, J. , & Sayal, K. (2014). Are self‐directed parenting interventions sufficient for externalising behaviour problems in childhood? A systematic review and meta‐analysis. European Child & Adolescent Psychiatry, 23, 1123–1137. 24842197 10.1007/s00787-014-0556-5

[jcpp14153-bib-0036] Thöne, A.‐K. , Görtz‐Dorten, A. , Altenberger, P. , Dose, C. , Geldermann, N. , Hautmann, C. , & Döpfner, M. (2020). Toward a dimensional assessment of externalizing disorders in children: Reliability and validity of a semi‐structured parent interview. Frontiers in Psychology, 11, 1840. 32849082 10.3389/fpsyg.2020.01840 PMC7396521

[jcpp14153-bib-0037] Thongseiratch, T. , Leijten, P. , & Melendez‐Torres, G.J. (2020). Online parent programs for children's behavioral problems: A meta‐analytic review. European Child & Adolescent Psychiatry, 29, 1555–1568. 31925545 10.1007/s00787-020-01472-0

[jcpp14153-bib-0038] Wolraich, M.L. , Hagan, J.F. , Allan, C. , Chan, E. , Davison, D. , Earls, M. , & Zurhellen, W. (2019). Clinical practice guideline for the diagnosis, evaluation, and treatment of attention‐deficit/hyperactivity disorder in children and adolescents. Pediatrics, 144, e20192528. 31570648 10.1542/peds.2019-2528 PMC7067282

