# Supplementary material for: Efficacy of guided and unguided web‐assisted self‐help for parents of children with attention‐deficit/hyperactivity disorder and oppositional defiant disorder: A three‐arm randomized controlled trial
Source: J Child Psychol Psychiatry. 2025 Mar 10;66(9):1320–32. doi: 10.1111/jcpp.14153 (PMC12350813; doi:10.1111/jcpp.14153)
Supplement: Supplementary file 1 — Table S1 Components of the web‐assisted self‐help intervention. Table S2. Description and psychometric properties of the outcome measures. Table S3. Sociodemographic and clinical characteristics at baseline (intention‐to‐treat sample). Table S4. Nonstudy treatments/treatment as usual. Table S5. Dropout analysis comparing families with and without available postassessment data (T3). Table S6. Descriptive statistics for the primary and secondary outcome measures (based on available data). Table S7. Group comparisons regarding the mean change from baseline (T1) to follow‐up (T4) based on mixed model repeated measures (MMRM) analyses: intention‐to‐treat sample. Table S8. Group comparisons regarding the mean change from baseline (T1) to postassessment (T3) and from baseline to follow‐up (T4) based on mixed model repeated measures (MMRM) analyses: per‐protocol sample (WASH: made use of at least 25% of the WASH intervention; WASH+S: additionally participated in at least 2 telephone consultations). Table S9. Group comparisons regarding the mean change from baseline (T1) to postassessment (T3) and from baseline to follow‐up (T4) based on mixed model repeated measures (MMRM) analyses: per‐protocol sample (WASH: made use of at least 40% of the WASH intervention; WASH+S: additionally participated in at least 3 telephone consultations). Table S10. Group comparisons regarding the mean DCL‐EXT change from baseline (T1) to postassessment (T3) based on mixed model repeated measures (MMRM) analyses: sensitivity analyses in the intention‐to‐treat sample. Table S11. Prediction of blinded clinician‐rated child externalizing symptoms (primary outcome) at T3 in the TAU condition: intention‐to‐treat sample. Table S12. Prediction of blinded clinician‐rated child externalizing symptoms (primary outcome) at T3 in the WASH+TAU condition: intention‐to‐treat sample. Table S13. Prediction of blinded clinician‐rated child externalizing symptoms (primary outcome) at T3 in the WASH+S+TAU condition: [file JCPP-66-1320-s001.pdf]

Online supplement for the article:

Efficacy of Guided and Unguided Web-Assisted Self-Help for Parents of Children with Attention-Deficit/Hyperactivity Disorder and Oppositional Defiant Disorder: A Three-Arm Randomized Controlled Trial

The first table (Table S1) of this online supplement describes the components and contents of the web-assisted self-help (WASH) intervention which was used in the present study. Table S2 comprises information on the measures used in the three-arm randomized controlled trial on the efficacy of the WASH intervention and on their psychometric properties. Table S3 displays baseline sociodemographic and clinical characteristics of the intention-to-treat sample considered for the analyses. Table S4 displays the utilization of non-study treatments in all study conditions. Table S5 refers to a comparison of families with and without available post-assessment data (i.e., "participants" vs. "dropouts") with regard to baseline sociodemographic and clinical characteristics. Table S6 contains descriptive statistics for the outcome measures in the three study conditions at the different assessment points. Table S7 presents the results of analyses of mixed models for repeated measures (MMRM analyses) comparing the three study conditions regarding the mean change from baseline to follow-up in the intention-to-treat sample. Tables S8 and S9 remaining tables display the results of MMRM analyses comparing the three study conditions in two differently defined per-protocol samples, including participants from the WASH conditions with either at least 25% treatment utilization (Table S8) or at least 40% treatment utilization (Table S9). Table S10 shows the results of sensitivity analyses, controlling for the use of pharmacological and non-pharmacological non-study treatments and analyzing the influence of diagnostic status (children with a formal diagnosis of an externalizing disorder vs. children without a formal

diagnosis). Tables S11 to S13 display the results of prediction analyses in the different study conditions.

Table S1

Components of the web-assisted self-help intervention

Module	(1) Dealing with Problem Behaviors[a]	(2) Psychoeducation	(3) Encouragement of Positive Parent-Child Interactions	(4) Self-Care for Parents
Submodules	(1) Definition of Individual Target Problems<br>(2) Psychoeducation about Coercive Parent-Child Interactions<br>(3) Reconsideration and Definition of Family Rules<br>(4) Effective Methods of Communicating Demands<br>(5) Positive Consequences of Following Rules<br>(6) Appropriate Negative Consequences of Breaking Rules<br>(7) Implementation of Reward Systems	(1) Symptoms of ADHD<br>(2) Comorbid Symptoms and Problems<br>(3) Causes of ADHD<br>(4) Developmental Course of Children with ADHD<br>(5) Treatment of ADHD<br>(6) Medication	(1) Assessing the Quality of the Parent-Child Relationship<br>(2) Identifying Positive Child Characteristics<br>(3) Implementing Play Times<br>(4) Encouraging the Child and Promoting the Child's Strengths	(1) Assessing Own Stress Level<br>(2) Own Strengths and Weaknesses<br>(3) Reconsidering and Improving Daily Structures<br>(4) Implementation of Positive Activities<br>(5) Dealing with Special Challenges

Note. [a]In module 1 "dealing with problem behaviors", seven typical problem situations which often occur in families of children with externalizing behavior problems are presented to the parents (tantrums, problems with homework, chaos in the children's room, frequent interruptions, media consumption, arguments between siblings, and hyperactivity and impulsivity at mealtimes). Following a seven-step approach (submodules), the parents are supported in analyzing the problem behaviors of their child and in developing solutions for these problem behaviors. To further illustrate the implementation of these steps, their application to the aforementioned typical problem situations is demonstrated in video vignettes.

Table S2

Description and psychometric properties of the outcome measures

Outcome	Rater	Measure	Description	Psychometric properties
ADHD and ODD symptoms	Blinded or unblinded clinician (depending on assessment point)	Diagnostic Checklist for Externalizing Behavior Disorders (DCL-EXT; Döpfner & Görtz-Dorten, 2017)	• Diagnostic checklist to assess symptoms of ADHD / ODD according to DSM-5 and ICD-10 criteria<br>• Composed of the 18 symptom-related items of the Diagnostic Checklist for Attention-Deficit/Hyperactivity Disorder (DCL-ADHD) and the 8-item ODD subscale of the Diagnostic Checklist for Disruptive Behavior Disorders (DCL-DBD)<br>• All items are rated on a 4-point Likert-type scale ranging from 0 (*not at all*) to 3 (*very much*)<br>• For the current study, the items were summarized in a total Externalizing Symptoms score (by averaging the item scores)<br>• To facilitate the ratings, a semi-structured interview guide (ILF-EXTERNAL) was used	• Internal consistency: Cronbach's α = .84 for the total score; α = .71 for the Inattention subscale and α = .87 for the Hyperactivity/Impulsivity subscale of the DCL-ADHD[1]; Cronbach's α = .60–.86 for the subscales and total scale of the DCL-SSV (Thöne et al., 2020)<br>• Good to excellent interrater reliability (intraclass coefficient ≥ .83 for the DCL-ADHD and ≥ .90 for the DCL-SSV; Thöne et al., 2020)<br>• Convergent and divergent validity of both the DCL-ADHD and the DCL-SSV (Thöne et al., 2020)<br>• Internal consistency in the current sample: Cronbach's α = .79 for the total Externalizing Symptoms score and α = .67–.78 for the DCL subscales
ADHD symptoms	Caregiver	Symptom Checklist for Attention-Deficit/Hyperactivity Disorder (SCL-ADHD; Döpfner & Görtz-Dorten, 2017)	• Rating scale to assess symptoms of ADHD according to DSM-5 and ICD-10 criteria<br>• 18 symptom-related items are rated on a 4-point Likert-type scale ranging from 0 (*not at all*) to 3 (*very much*)<br>• Two symptom-related subscales: Inattention, Hyperactivity/Impulsivity<br>• 5 additional items to assess ADHD-related impairment	• Factorial validity (Döpfner & Görtz-Dorten, 2017; Erhart et al., 2008)<br>• Internal consistency: Cronbach's α > .80 for all subscales and the total score (Döpfner & Görtz-Dorten, 2017; Erhart et al., 2008)<br>• Internal consistency in the current sample: Cronbach's α = .84–.89 for the SCL-ADHD subscales and total scale, Cronbach's α = .66 for the Functional Impairment scale
Symptoms of disruptive behavior disorders	Caregiver	ODD scale of the Symptom Checklist for Disruptive Behavior Disorders (SCL-	• The SCL-DBD assesses symptoms of disruptive behavior disorders according to ICD-10 and DSM-V as well as characteristics of limited prosocial emotions	• Internal consistency: Cronbach's α > .80 for all subscales and the total score of the SCL-DBD (Döpfner &

Table S2

Description and psychometric properties of the outcome measures

Outcome	Rater	Measure	Description	Psychometric properties
		DBD; Döpfner & Görtz-Dorten, 2017)	• Only the 8-item ODD scale was considered for the current analyses<br>• All items are rated on a 4-point Likert-type scale ranging from 0 (*not at all*) to 3 (*very much*)	Görtz-Dorten, 2017; Görtz-Dorten et al., 2014)<br>• Factorial, convergent and divergent validity (Döpfner & Görtz-Dorten, 2017; Görtz-Dorten et al., 2014)<br>• Internal consistency of the SCL-DBD ODD subscale in the current sample: Cronbach's α = .88
Broad spectrum of child behavioral and emotional problems	Caregiver	German version of the Child Behavior Checklist for Ages 6–18 (CBCL 6–18; Achenbach & Rescorla, 2001; Döpfner et al., 2014)	• Rating scale to assess behavioral and emotional problems<br>• 120 items are rated on a 3-point scale from 0 (*not true*) to 2 (*very true or often true*)<br>• Items can be aggregated to eight syndrome scales as well as three superordinate scales: Internalizing Problems, Externalizing Problems, Total Problems<br>• For the current analyses, only the Internalizing Problems scale and Externalizing Problems scale were used	• Cross-cultural factorial validity (Ivanova et al., 2007)<br>• Internal consistency: CBCL total score: Cronbach's α =.93 in both a community and a clinical sample; CBCL Internalizing Problems: Cronbach's α =.82 in a community sample and Cronbach's α =.85 in a clinical sample; CBCL Externalizing Problems: Cronbach's α =.88 in a community sample and Cronbach's α =.91 in a clinical sample (Döpfner et al., 2014)<br>• Internal consistency in the current sample: Cronbach's α = .87 for the Internalizing Problems scale and Cronbach's α = .89 for the Externalizing Problems scale
Quality of life	Caregiver	German questionnaire for measuring health-related quality of life in children and adolescents (KIDSCREEN-10; KIDSCREEN Group Europe, 2006)	• Rating scale to assess health-related quality of life<br>• 10 items are rated on a 5-point Likert-type scale ranging from 1 (*never/not at all*) to 5 (*very much/always*)<br>• Items can be summarized in a total score	• Internal consistency (Ravens-Sieberer et al., 2010)<br>• Test-retest reliability (Ravens-Sieberer et al., 2010)<br>• Discriminant validity (KIDSCREEN-10 discriminates well between children with emotional and behavioral problems and normal

Table S2

Description and psychometric properties of the outcome measures

Outcome	Rater	Measure	Description	Psychometric properties
				controls; Ravens-Sieberer et al., 2010)<br>• Internal consistency in the current sample: Cronbach's α = .74
Positive and negative parenting practices	Caregiver	Assessment Scale of Positive and Negative Parenting Behavior (FPNE; Holas et al., 2024; Imort et al., 2014)	• 38 items rated on a 4-point Likert-type scale ranging from 1 (*never*) to 4 (*very often*)<br>• Items can be summarized in two subscales (Positive Parenting: 21 items, Negative Parenting:17 items)	• Internal consistency (Imort et al., 2014)<br>• Current sample: α = .71 for the Negative Parenting subscales and α = .88 for the Positive Parenting subscale
Parental symptoms depression, anxiety and stress	Caregiver	Depression Anxiety Stress Scales (DASS; S. H. Lovibond & Lovibond, 1995; P. F. Lovibond & Lovibond, 1995; Nilges & Essau, 2015)	• 42 items rated on a 4-point Likert-type scale ranging from 0 (*did not apply to me at all*) to 3 (*applied to me very much, or most of the time*)<br>• Items can be aggregated to three subscales (Depression, Anxiety, Stress) and a total score; only the total score was considered for the current analyses	• Factorial validity (P. F. Lovibond & Lovibond, 1995)<br>• Internal consistency (Cronbach's α ≥ .81; P. F. Lovibond & Lovibond, 1995)<br>• Convergent validity (P. F. Lovibond & Lovibond, 1995)<br>• Current sample: α = .95 for the total scale
Satisfaction with the intervention	Caregiver	Client Satisfaction Questionnaire adapted to Internet-based interventions (CSQ-I; Boß et al., 2016)	• 8 items rated on a 4-point scale ranging from 1 (*does not apply to me*) to 4 (*does totally apply to me*)	• Factorial validity (Boß et al., 2016)<br>• Internal consistency (Boß et al., 2016)<br>• Construct validity (Boß et al., 2016)<br>• Current sample: Cronbach's α = .94

Table S3

Sociodemographic and clinical characteristics at baseline (intention-to-treat sample)

Variable	Condition	*n*	*M*/%	*SD*	*range*	Test statistics[1]	*p*
Child							
Age (years)	Total sample	431	9.35	1.68	6.00 – 12.99		
	TAU	147	9.38	1.59	6.14 – 12.99		
	WASH	141	9.31	1.71	6.00 – 12.97	*F*=.088	.916
	WASH+S	143	9.37	1.74	6.00 – 12.92		
Sex						χ^2=.271	.873
Sex (male)	Total sample	351	81.4%				
	TAU	120	81.6%				
	WASH	113	80.1%				
	WASH+S	118	82.5%				
Sex (female)	Total sample	80	18.6%				
	TAU	27	18.4%				
	WASH	28	19.9%				
	WASH+S	25	17.5%				
DCL-EXT Total score	Total sample	431	1.34	0.35	1.30 - 1.37		
	TAU	147	1.33	0.35	1.27 – 1.38		
	WASH	141	1.37	0.33	1.31 – 1.42	*F*=.656	.520
	WASH+S	143	1.32	0.37	1.26 – 1.38		
Comorbidity (CBCL total score)	Total sample	420	59.34	22.50	6 – 130		
	TAU	144	59.38	22.81	19 – 130		
	WASH	135	59.59	21.90	16 – 115	*F*=.019	.981
	WASH+S	141	59.06	22.90	6 – 118		
Quality of life (KIDSCREEN total score)	Total sample	420	36.81	4.94	20 – 49		
	TAU	144	37.17	4.81	23 – 48		
	WASH	135	36.58	5.17	21 – 49	*F*=.610	.544
	WASH+S	141	36.65	4.q85	20 – 48		
Functional Impairment (SCL-ADHD Functional Impairment scale)	Total sample	420	1.72	0.63	0.25 – 3.00		
	TAU	144	1.66	0.61	0.25 – 3.00		
	WASH	135	1.80	0.63	0.40 – 3.00	*F=2*.025	.133
	WASH+S	141	1.69	0.63	0.25 – 3.00		
Functional Impairment (SCL-DBD Functional Impairment scale)	Total sample	431	1.51	0.62	0.00 – 3.00		
	TAU	147	1.50	0.63	0.00 – 3.00		
	WASH	141	1.54	0.65	0.00 – 3.00	*F*=.229	.795
	WASH+S	143	1.49	0.58	0.00 – 3.00		
Caregiver							
Sex						χ^2=.363	.834
Sex (female)	Total sample	392	91.0%				
	TAU	132	89.8%				
	WASH	129	91.5%				
	WASH+S	131	91.6%				
Sex (male)	Total sample	39	9.0%				
	TAU	15	10.2%				
	WASH	12	8.5%				
	WASH+S	12	8.4%				
Positive parenting behavior (FPNE positive parenting)	Total sample	420	3.02	0.35	1.90 – 3.86		
	TAU	144	3.02	0.39	1.90 – 3.86		
	WASH	135	3.02	0.33	2.19 – 3.71	*F*=.010	.990
	WASH+S	141	3.02	0.32	2.29 – 3.81		
Negative parenting behavior (FPNE negative parenting)	Total sample	420	1.93	0.29	1.24 – 2.82		
	TAU	144	1.93	0.29	1.29 – 2.76	*F*=.085	.918
	WASH	135	1.93	0.29	1.29 – 2.71		

	WASH+S	141	1.94	0.28	1.24 – 2.82		
Parental internalizing symptoms (DASS total score)	Total sample	420	0.62	0.41	0 – 2.26		
	TAU	144	0.65	0.41	0 – 2.17		
	WASH	135	0.60	0.37	0 – 1.83	*F*=.514	.599
	WASH+S	141	0.62	0.43	0 – 2.26		
Educational level[2]		Total	TAU	WASH	WASH+S		
Primary and lower secondary level of education (ISCED level 1 and 2)[3]		12.9%	11.1%	12.6%	14.9%		
Upper secondary level of education (ISCED level 3B)[4]		27.6%	29.9%	27.4%	25.5%		
Upper secondary level of education (ISCED level 3A) and post-secondary non-tertiary (ISCED level 4)[5]		19.0%	15.3%	17.0%	24.8%	*H*=0.805	.669
First stage of tertiary education (ISCED level 5B)[6]		12.9%	18.8%	11.1%	8.5%		
First stage (ISCED level 5A) or second stage of tertiary education (ISCED level 6)[7]		27.6%	25.0%	31.9%	26.2%		

Note. TAU = treatment as usual, WASH = web-assisted self-help, WASH+S = web-assisted self-help plus telephone-based support; DCL-EXT = Diagnostic Checklist for Externalizing Behavior Disorders, CBCL = Child Behavior Checklist, KIDSCREEN = German questionnaire on health-related quality of life, SCL-ADHD = Symptom Checklist for Attention-Deficit/Hyperactivity Disorder, SCL-DBD = Symptom Checklist for Disruptive Behavior Disorders, FPNE = Assessment Scale of Positive and Negative Parenting Behavior, DASS = Depression Anxiety Stress Scales; ISCED = International Standard Classification of Education (OECD, 1997); *n* = sample size, *m* = Mean, *SD* = standard deviation, *p* = significance value.

[1] test statistics for between-group comparisons: binary outcomes – binomial test, categorical outcomes – χ^2 test, ordered categorical outcomes – Kruskal-Wallis *H*-test, continuous outcomes – univariate analyses of covariance (ANOVA, *F*-test).

[2] educational level according to the International Standard Classification of Education (OECD, 1997)

[3] primary school, special school, lower secondary school diploma (German: Haupt-/Volksschulabschluss, Realschulabschluss, polytechnische Oberschule)

[4] basic vocational training year, vocational schools, specialized vocational schools, commercial school

[5] vocational high school, specialized vocational high school, specialized vocational schools: qualification for ISCED 5A, “Kollegschulen”: qualification for ISCED 5A, upper secondary schools (German diplomas: Fachhochschulreife, Hochschulreife)

[6] trade and technical school (German diplomas: Meister/Techniker, Fachschulabschluss), health sector school, vocational academy

[7] university, German “Fachhochschule”, doctoral studies (diplomas: Diplom (FH). university diploma, Bachelor, Master, PhD)

Table S4
Non-study treatments/treatment as usual

	T1								Group comparison		
	Total (*n* = 420)		WASH+S +TAU (*n* = 141)		WASH+ TAU (*n* = 135)		TAU (*n* = 144)				
	n	%	*n*	%	*n*	%	*n*	%	χ^2	*df*	*p*
Psychotherapy	109	26.0	43	30.5	30	22.2	36	25.0	2.56	2	.273
Ergotherapy/occupational therapy	119	28.3	42	29.8	39	28.9	38	26.4	0.44	2	.809
Physiotherapy	11	2.6	5	3.5	3	2.2	3	2.1	0.72	2	.744
Parent training	15	3.6	7	5.0	4	3.0	4	2.8	1.20	2	.608
Self-help group for parents	19	4.5	6	4.3	8	5.9	5	3.5	1.01	2	.612
Internet-based self-help for parents	11	2.6	1	0.7	4	3.0	6	4.2	3.43	2	.173
Others	39	9.3	17	12.1	13	9.6	9	6.3	2.88	2	.237
At least one non-pharmacological treatment	238	56.7	88	62.4	74	54.8	76	52.8	2.97	2	.228
Medication	230	54.8	75	53.2	72	53.3	83	57.6	0.73	2	.701
No non-study treatment	91	21.7	29	20.6	34	25.2	28	19.4	1.50	2	.478
	T2								Group comparison		
	Total (*n* = 299)		WASH+S +TAU (n = 97)		WASH+ TAU (n = 93)		TAU (n = 109)				
	n	%	*n*	%	*n*	%	*n*	%	χ^2	*df*	*p*
Psychotherapy	84	28.1	33	34.0	25	26.9	26	23.9	2.73	2	.255
Ergotherapy/occupational therapy	78	26.1	23	23.7	23	24.7	32	29.4	0.98	2	.634
Physiotherapy	7	2.3	1	1.0	4	4.3	2	1.8	2.41	2	.309
Parent training	42	14.0	22	22.7	16	17.2	4	3.7	16.48	2	< .001
Self-help group for parents	17	5.7	6	6.2	5	5.4	6	5.5	0.07	2	> .999
Internet-based self-help for parents	74	24.8	27	28.1	44	47.3	3	2.8	54.20	2	< .001
Others	23	7.8	9	9.4	6	6.5	8	7.4	0.57	2	.797
At least one non-pharmacological treatment	204	68.5	72	75.0	72	77.4	60	55.0	14.44	2	< .001
At least one non-pharmacological treatment – internet-based self-help excluded	180	60.4	65	67.7	56	60.2	59	54.1	3.94	2	.145
Medication	188	63.1	61	63.5	59	63.4	68	62.4	0.04	2	.977
No non-study treatment	39	13.1	13	13.7	7	7.5	19	17.4	4.35	2	.114
	T3								Group comparison		
	Total (*n* = 260)		WASH+S +TAU (*n* = 82)		WASH+ TAU (*n* = 78)		TAU (*n* = 100)				
	n	%	*n*	%	*n*	%	*n*	%	χ^2	*df*	*p*
Psychotherapy	71	27.3	28	34.1	17	21.8	26	26.0	3.21	2	.203
Ergotherapy/occupational therapy	57	21.8	20	24.1	18	23.1	19	19.0	0.79	2	.702
Physiotherapy	7	2.7	3	3.6	2	2.6	2	2.0	0.46	2	.891
Parent training	32	12.3	17	20.7	7	9.0	8	8.0	7.91	2	.020
Self-help group for parents	18	6.9	5	6.1	6	7.7	7	7.0	0.16	2	.953
Internet-based self-help for parents	62	23.8	24	29.3	31	39.7	7	7.0	27.81	2	< .001
Others	24	9.2	5	6.1	12	15.4	7	7.0	5.08	2	.079

At least one non-pharmacological treatment	160	61.3	59	71.1	54	69.2	47	47.0	14.04	2	< .001
At least one non-pharmacological treatment – internet-based self-help excluded	145	55.6	55	66.3	45	57.7	45	45.0	8.51	2	.015
Medication	169	65.0	56	68.3	50	64.1	63	63.0	0.59	2	.767
No non-study treatment	43	16.5	10	12.0	12	15.4	21	21.0	2.74	2	.256

	T4								Group comparison		
	Total (n = 246)		WASH+S +TAU (n = 77)		WASH+ TAU (n = 79)		TAU (= 90)				
	n	%	*n*	%	*n*	%	*n*	%	χ^2	*df*	*p*
Psychotherapy	66	26.8	28	36.4	14	17.7	24	26.7	6.91	2	.032
Ergotherapy/occupational therapy	45	18.2	12	15.8	18	22.2	15	16.7	1.32	2	.536
Physiotherapy	9	3.5	4	4.9	2	2.4	3	3.3	0.80	2	.704
Parent training	25	9.7	12	14.6	8	9.3	5	5.6	4.06	2	.124
Self-help group for parents	19	7.4	7	8.5	5	5.9	7	7.8	0.46	2	.833
Internet-based self-help for parents	40	15.6	17	20.5	21	24.2	2	2.3	18.49	2	< .001
Others	34	13.7	13	16.5	11	13.3	10	11.5	0.88	2	.641
At least one non-pharmacological treatment	161	63.4	60	73.2	55	65.5	46	52.3	8.22	2	.016
At least one non-pharmacological treatment – internet-based self-help excluded	142	55.9	53	64.6	44	52.4	45	51.1	3.77	2	.153
Medication	166	64.1	55	66.3	52	60.5	59	65.5	0.75	2	.709
No non-study treatment	37	14.5	10	12.0	13	15.3	14	15.9	0.59	2	.773

Note. TAU = treatment as usual, WASH = web-assisted self-help, WASH+S = web-assisted self-help plus telephone-based support.

Table S5

Dropout analysis comparing families with and without available post-assessment data (T_3)

Variable	Category	Condition	Participants (*n*=333) *M/n*	*(LB; UB)*	Dropout (*n*=98) *M/n*	*(LB; UB)*	*p*[1]
Child age at baseline (years)		TAU	9.4	(9.2; 9.7)	9.1	(8.6; 9.7)	.322
		WASH	9.4	(9.0; 9.7)	9.2	(8.6; 9.7)	.558
		WASH+S	9.4	(9.1; 9.7)	9.3	(8.6; 9.9)	.718
Child sex	female	TAU	20 (17%)		7 (22%)		.562
		WASH	18 (17%)		10 (29%)		.109
		WASH+S	18 (16%)		7 (22%)		.458
Age of participating caregiver at baseline (years)		TAU	41.1	(40.1; 42.0)	39.4	(37.3; 41.5)	.121
		WASH	41.9	(40.7; 43.1)	40.3	(38.4; 42.2)	.182
		WASH+S	42.4	(41.3; 43.5)	40.9	(38.8; 43.0)	.201
Sex of participating caregiver	female	TAU	106 (92%)		26 (81%)		.071
		WASH	98 (92%)		31 (91%)		.940
		WASH+S	103 (93%)		28 (88%)		.341
Referring healthcare provider (pediatrician vs. child psychiatrist)	child psychiatrist	TAU	45 (39%)		13 (41%)		.878
		WASH	40 (37%)		12 (35%)		.826
		WASH+S	50 (45%)		12 (38%)		.448
Supply area (urban vs. rural)	rural	TAU	18 (16%)		7 (22%)		.407
		WASH	19 (18%)		3 (9%)		.211
		WASH+S	17 (15%)		9 (28%)		.098
Highest educational level achieved by participating caregiver[2]	ISCED level 1 and 2[3]	TAU	13 (11%)		3 (10%)		.466
	ISCED level 3B[4]		20 (26%)		13 (43%)		
	ISCED level 3A and 4[5]		18 (16%)		4 (13%)		
	ISCED level 5B[6]		22 (19%)		5 (17%)		
	ISCED level 5A and 6[7]		31 (27%)		5 (17%)		
	ISCED level 1 and 2[3]	WASH	13 (12%)		4 (13%)		.693
	ISCED level 3B[4]		27 (26%)		10 (32%)		
	ISCED level 3A and 4[5]		16 (15%)		7 (23%)		
	ISCED level 5B[6]		12 (12%)		3 (10%)		
	ISCED level 5A and 6[7]		36 (35%)		7 (23%)		
	ISCED level 1 and 2[3]	WASH+S	14 (13%)		7 (23%)		.358
	ISCED level 3B[4]		27 (24%)		9 (30%)		
	ISCED level 3A and 4[5]		31 (28%)		4 (14%)		
	ISCED level 5B[6]		9 (8%)		3 (10%)		
	ISCED level 5A and 6[7]		30 (27%)		7 (23%)		
Participating caregiver currently employed[8]	Yes	TAU	99 (87%)		19 (63%)		.003
		WASH	85 (82%)		29 (94%)		.111
		WASH+S	97 (87%)		25 (83%)		.564
Child externalizing symptoms (DCL total score) at baseline		TAU	1.49	(1.42; 1.56)	1.52	(1.38; 1.66)	.721
		WASH	1.56	(1.49; 1.63)	1.55	(1.42; 1.67)	.880
		WASH+S	1.51	(1.44; 1.58)	1.43	(1.27; 1.60)	.338
Child ADHD symptoms (SCL-ADHD total score) at baseline		TAU	1.71	(1.61; 1.80)	1.87	(1.69; 2.04)	.139
		WASH	1.83	(1.74; 1.92)	1.82	(1.61; 2.03)	.909
		WASH+S	1.72	(1.62; 1.82)	1.74	(1.51; 1.97)	.842
Child broadband comorbid symptoms (CBCL total score) at baseline		TAU	58.35	(54.11; 62.59)	63.27	(54.84; 71.69)	.295
		WASH	60.96	(56.65; 65.27)	54.97	(47.39; 62.55)	.182
		WASH+S	59.11	(54.95; 63.27)	58.90	(49.19; 68.61)	.965
Child quality of life (KIDSCREEN total score) at baseline		TAU	37.15	(36.28; 38.02)	37.27	(35.30; 39.23)	.906
		WASH	36.34	(35.31; 37.36)	37.39	(35.60; 39.17)	.323
		WASH+S	36.40	(35.49; 37.30)	37.60	(35.75; 39.45)	.229
Positive parenting behavior		TAU	3.00	(2.93; 3.08)	3.07	(2.93; 3.22)	.385

Note. Column header "Mean (95% confidence interval)/ Frequency (%)" spans the Participants and Dropout columns.

Variable	Category	Condition	Mean (95% confidence interval)/ Frequency (%) Participants (*n*=333) *M/n*	*(LB; UB)*	Dropout (*n*=98) *M/n*	*(LB; UB)*	*p*[1]
(FPNE positive parenting scale) at		WASH	3.01	(2.95; 3.07)	3.06	(2.91; 3.21)	.458
baseline		WASH+S	3.02	(2.96; 3.08)	3.03	(2.93; 3.13)	.915
Negative parenting behavior		TAU	1.93	(1.88; 1.99)	1.90	(1.78; 2.02)	.589
(FPNE negative parenting scale) at		WASH	1.94	(1.88; 2.00)	1.90	(1.79; 2.01)	.528
baseline		WASH+S	1.95	(1.89; 2.00)	1.93	(1.83; 2.02)	.725
Parental internalizing symptoms		TAU	0.61	(0.54; 0.69)	0.77	(0.61; 0.93)	.057
(DASS total score) at baseline		WASH	0.61	(0.54; 0.68)	0.56	(0.42; 0.70)	.525
		WASH+S	0.63	(0.55; 0.71)	0.59	(0.41; 0.77)	.680

Note. TAU = treatment as usual, WASH = web-assisted self-help, WASH+S= web-assisted self-help and additional support, DCL = Diagnostic Checklist for Externalizing Behavior Disorders, SCL-ADHD = Symptom Checklist for Attention-Deficit/Hyperactivity Disorder, CBCL = Child Behavior Checklist, KIDSCREEN = questionnaire to assess health-related quality of life, FPNE = Assessment Scale of Positive and Negative Parenting Behavior, DASS = Depression Anxiety Stress Scales, ISCED = International Standard Classification of Education; *M* = mean; *n* = number of cases; (LB; UB) = lower and upper bound of 95% confidence interval.

[1] test statistics for between-group comparisons: binary outcomes – binomial test, categorical outcomes – χ^2 test, ordered categorical outcomes – Kruskal-Wallis H-test, continuous outcomes – univariate analyses of covariance (ANOVA, *F*-test).

[2] educational level according to the International Standard Classification of Education (OECD, 1997)

[3] primary and lower secondary level of education: primary school, special school, lower secondary school diploma (German: Haupt-/Volksschulabschluss, Realschulabschluss, polytechnische Oberschule)

[4] upper secondary level of education: basic vocational training year, vocational schools, specialized vocational schools, commercial school

[5] upper secondary level of education and post-secondary non-tertiary: vocational high school, specialized vocational high school, specialized vocational schools: qualification for ISCED 5A, “Kollegschulen”: qualification for ISCED 5A, upper secondary schools (German diplomas: Fachhochschulreife, Hochschulreife, Abitur)

[6] first stage of tertiary education: trade and technical school (German diplomas: Meister/Techniker, Fachschulabschluss), health sector school, vocational academy

[7] first stage or second stage of tertiary education: university, German “Fachhochschule”, doctoral studies (diplomas: Fachhochschulabschluss, Diplom (FH), university diploma, Bachelor, Master, PhD)

[8] Four missing values in the participants and seven missing values in the dropouts.

Table S6

Descriptive statistics for the primary and secondary outcome measures (based on available data)

Variable	Condition	T1			T2			T3			T4		
		n	*M*	*SD*	*n*	*M*	*SD*	*n*	*M*	*SD*	*n*	*M*	*SD*
DCL-EXT Total score	TAU	147	1.50	0.38	116	1.26	0.44	105	1.14	0.46	98	1.07	0.44
	WASH	141	1.55	0.36	112	1.23	0.39	96	1.17	0.42	90	1.12	0.46
	WASH+S	143	1.49	0.40	118	1.14	0.41	106	1.03	0.46	102	1.03	0.47
SCL-ADHD Total score	TAU	144	1.74	0.53	112	1.55	0.55	101	1.46	0.55	90	1.39	0.58
	WASH	135	1.83	0.47	94	1.53	0.51	84	1.46	0.51	82	1.37	0.55
	WASH+S	141	1.72	0.56	101	1.44	0.53	83	1.41	0.57	83	1.29	0.60
SCL-DBD ODD scale	TAU	144	1.49	0.65	106	1.37	0.68	101	1.35	0.73	91	1.26	0.71
	WASH	135	1.54	0.66	90	1.36	0.61	84	1.26	0.71	86	1.23	0.72
	WASH+S	141	1.46	0.72	99	1.23	0.61	83	1.18	0.67	85	1.11	0.64
CBCL Internalizing symptoms	TAU	144	12.66	8.30	109	11.12	8.10	100	10.53	8.13	90	9.86	7.14
	WASH	135	12.07	7.74	93	9.90	7.30	78	10.46	7.76	79	10.01	7.65
	WASH+S	141	12.45	8.32	97	9.99	7.24	83	9.45	6.70	77	8.39	6.43
CBCL Externalizing symptoms	TAU	144	18.13	8.54	109	15.24	8.14	100	15.29	8.80	90	13.47	9.38
	WASH	135	18.69	8.58	93	15.99	8.78	78	14.83	8.87	79	14.16	8.57
	WASH+S	141	18.60	9.10	97	15.90	8.62	83	15.05	9.13	77	14.34	9.62
SCL-ADHD Functional Impairment	TAU	144	1.66	0.61	107	1.45	0.66	100	1.16	0.43	89	1.10	0.47
	WASH	135	1.80	0.63	92	1.47	0.61	77	1.14	0.43	82	1.04	0.51
	WASH+S	141	1.69	0.63	97	1.40	0.60	83	1.11	0.47	80	0.94	0.44
KIDSCREEN Total score	TAU	144	37.17	4.81	109	37.48	5.46	100	37.49	5.66	90	37.59	6.04
	WASH	135	36.58	5.17	92	36.21	5.09	78	37.19	4.73	86	36.62	4.88
	WASH+S	141	36.65	4.85	97	37.33	4.72	83	36.89	4.88	82	36.59	5.63
FPNE Positive parenting	TAU	144	3.02	0.39	109	2.98	0.39	100	3.01	0.41	90	3.04	0.39
	WASH	135	3.02	0.33	93	3.03	0.34	78	3.03	0.40	86	2.97	0.45
	WASH+S	141	3.02	0.32	97	3.04	0.34	81	3.02	0.30	83	2.90	0.44
FPNE Negative parenting	TAU	144	1.93	0.29	109	1.90	0.29	100	1.86	0.29	90	1.83	0.30
	WASH	135	1.93	0.29	93	1.84	0.27	78	1.85	0.33	86	1.83	0.30
	WASH+S	141	1.94	0.28	97	1.80	0.24	82	1.81	0.27	83	1.81	0.26
DASS Total score	TAU	144	0.65	0.41	109	0.56	0.43	100	0.51	0.42	90	0.50	0.43
	WASH	135	0.60	0.37	93	0.56	0.38	78	0.58	0.47	81	0.51	0.41
	WASH+S	141	0.62	0.43	97	0.47	0.42	83	0.50	0.52	77	0.45	0.40

Note. TAU = treatment as usual, WASH = web-assisted self-help, WASH+S= web-assisted self-help and additional support; DCL = Diagnostic Checklist for Externalizing Behavior Disorders, SCL-ADHD = Symptom Checklist for Attention-Deficit/Hyperactivity Disorder, CBCL = Child Behavior Checklist, KIDSCREEN = questionnaire to assess health-related quality of life, FPNE = Assessment Scale of Positive and Negative Parenting Behavior, DASS = Depression Anxiety Stress Scales; T1 = baseline, T2 = interim assessment, T3 = post-assessment, T4 = follow-up; *n* = sample size, *M* = mean, *S*D = standard deviation.

Table S7

Group comparisons regarding the mean change from baseline (T_1) to follow-up (T_4) based on mixed model repeated measures (MMRM) analyses: intention-to-treat sample

		Marginal means									Total effect	Pairwise comparisons																	
	Condition	T2			T3			T4				TAU vs. WASH						TAU vs. WASH+S						WASH vs. WASH+S					
Variable	)	*M*	*(LB;*	*UB)*	*M*	*(LB;*	*UB)*	*M*	*(LB;*	*UB)*	*p*	n_{TAU}	n_{WASH}	*d*	*(LB;*	*UB)*	p_d	n_{TAU}	n_{WASH+S}	*d*	*(LB;*	*UB)*	p_d	n_{WASH}	n_{WASH+S}	*d*	*(LB;*	*UB)*	p_d
	TAU	-0.24	-0.30	-0.18	-0.36	-0.42	-0.29	-0.42	-0.49	-0.34																			
DCL-EXT	WASH	-0.30	-0.35	-0.24	-0.37	-0.44	-0.30	-0.43	-0.51	-0.36	.396	126	124	-0.03	-0.28	0.21	.792	126	121	-0.16	-0.41	0.09	.198						
Total score	WASH+S	-0.38	-0.43	-0.32	-0.48	-0.54	-0.41	-0.49	-0.56	-0.41														124	121	-0.13	-0.38	0.12	.319
SCL-ADHD	TAU	-0.17	-0.25	-0.10	-0.27	-0.35	-0.20	-0.34	-0.43	-0.25																			
Total score	WASH	-0.25	-0.33	-0.17	-0.35	-0.43	-0.27	-0.48	-0.58	-0.39	.092	125	108	-0.27	-0.53	-0.02	.037	125	116	-0.20	-0.45	0.06	.130	108	116	0.08	-0.18	0.34	.557
	WASH+S	-0.29	-0.36	-0.21	-0.33	-0.40	-0.25	-0.44	-0.53	-0.35																			
SCL-DBD	TAU	-0.07	-0.15	0.01	-0.14	-0.24	-0.04	-0.23	-0.33	-0.12																			
ODD scale	WASH	-0.13	-0.22	-0.04	-0.23	-0.33	-0.12	-0.32	-0.42	-0.21	.095	125	107	-0.15	-0.41	0.11	.252	125	116	-0.28	-0.53	-0.03	.032	107	116	-0.13	-0.39	0.13	.331
	WASH+S	-0.22	-0.30	-0.14	-0.31	-0.41	-0.20	-0.39	-0.50	-0.28																			
CBCL	TAU	-1.73	-2.62	-0.84	-2.10	-3.06	-1.13	-2.74	-3.77	-1.71																			
Internalizing	WASH	-2.17	-3.13	-1.20	-1.84	-2.92	-0.77	-2.98	-4.10	-1.86	.651	119	105	-0.04	-0.30	0.22	.759	119	111	-0.12	-0.38	0.14	.362	105	111	-0.08	-0.35	0.19	.561
symptoms	WASH+S	-2.40	-3.34	-1.47	-3.41	-4.45	-2.38	-3.45	-4.55	-2.34																			
CBCL	TAU	-2.46	-3.42	-1.50	-2.70	-3.76	-1.63	-4.66	-5.90	-3.42																			
Externalizing	WASH	-2.19	-3.23	-1.14	-3.50	-4.69	-2.30	-4.62	-5.97	-3.27	.887	119	105	0.01	-0.26	0.27	.963	119	111	-0.05	-0.31	0.20	.683	105	111	-0.06	-0.33	0.21	.662
symptoms	WASH+S	-2.91	-3.92	-1.90	-4.34	-5.49	-3.20	-5.04	-6.37	-3.72																			
SCL-ADHD	TAU	-0.23	-0.32	-0.14	-0.55	-0.61	-0.48	-0.61	-0.70	-0.53																			
Functional	WASH	-0.28	-0.38	-0.18	-0.62	-0.69	-0.54	-0.74	-0.83	-0.65	.030	119	104	-0.27	-0.54	-0.01	.043	119	111	-0.32	-0.58	-0.06	.015	104	111	-0.05	-0.32	0.22	.710
Impairment	WASH+S	-0.30	-0.40	-0.20	-0.62	-0.70	-0.55	-0.77	-0.85	-0.68																			
KIDSCREEN	TAU	0.65	-0.17	1.47	0.72	-0.19	1.62	0.92	-0.07	1.90																			
Total score	WASH	-0.12	-1.02	0.78	0.66	-0.37	1.68	0.04	-0.99	1.07	.337	119	105	-0.16	-0.42	0.10	.232	119	110	-0.17	-0.43	0.08	.187	105	110	-0.02	-0.28	0.25	.904
	WASH+S	0.85	-0.02	1.71	0.84	-0.14	1.82	-0.05	-1.08	0.99																			
FPNE	TAU	-0.02	-0.06	0.02	0.01	-0.03	0.06	0.02	-0.05	0.09																			
Positive	WASH	0.02	-0.02	0.07	0.00	-0.05	0.06	-0.06	-0.13	0.01	.087	119	105	-0.21	-0.47	0.05	.116	119	111	-0.28	-0.54	-0.02	.036	105	111	-0.07	-0.34	0.20	.609
parenting	WASH+S	0.05	0.00	0.09	0.03	-0.02	0.09	-0.08	-0.16	-0.01																			
FPNE	TAU	-0.03	-0.07	0.01	-0.08	-0.12	-0.03	-0.10	-0.14	-0.05																			
Negative	WASH	-0.08	-0.12	-0.03	-0.08	-0.12	-0.03	-0.11	-0.16	-0.07	.751	119	105	-0.06	-0.33	0.20	.637	119	111	-0.10	-0.36	0.16	.458	105	111	-0.04	-0.30	0.23	.795
parenting	WASH+S	-0.13	-0.17	-0.09	-0.13	-0.17	-0.08	-0.12	-0.17	-0.08																			
DASS Total	TAU	-0.05	-0.11	0.00	-0.09	-0.16	-0.02	-0.11	-0.17	-0.04																			
score	WASH	-0.04	-0.10	0.03	-0.02	-0.10	0.05	-0.10	-0.17	-0.03	.828	119	105	0.03	-0.23	0.30	.803	119	111	-0.05	-0.31	0.21	.706	105	111	-0.08	-0.35	0.18	.545
	WASH+S	-0.11	-0.17	-0.05	-0.13	-0.20	-0.05	-0.13	-0.20	-0.06																			

Note. TAU = treatment as usual, WASH = web-assisted self-help, WASH+S= web-assisted self-help and additional support via telephone, DCL-EXT = Diagnostic Checklist for Externalizing Behavior Disorders, SCL-ADHD = Symptom Checklist for Attention-Deficit/Hyperactivity Disorder, SCL-DBD = Symptom Checklist for Disruptive Behavior Disorders, ODD = Oppositional Defiant Disorder, CBCL = Child Behavior Checklist, KIDSCREEN = questionnaire to assess health-related quality of life, FPNE = Assessment

Scale of Positive and Negative Parenting Behavior, DASS = Depression Anxiety Stress Scales, T1 = baseline, T2 = interim assessment, T3 = post-assessment, T4 = follow-up.

M = mean, LB = lower bound, UB = upper bound, *p* = significance value, *n* = sample size, *d* = Cohen’s *d* (effect size).

Table S8

Group comparisons regarding the mean change from baseline (T_1) to post-assessment (T_3) and from baseline to follow-up (T_4) based on mixed model repeated measures (MMRM) analyses: per-protocol sample (WASH: made use of at least 25% of the WASH intervention; WASH+S: additionally participated in at least 2 telephone consultations)

		Marginal means									Total effect	Pairwise comparisons																	
		T2			T3			T4				TAU vs. WASH						TAU vs. WASH+S						WASH vs. WASH+S					
Variable	Condition	*M*	(LB;	UB)	*M*	(LB;	UB)	*M*	(LB;	UB)	*p*	n_{TAU}	n_{WASH}	*d*	(LB;	UB)	p_d	n_{TAU}	n_{WASH+S}	*d*	(LB;	UB)	p_d	n_{WASH}	n_{WASH+S}	*d*	(LB;	UB)	p_d
Change from baseline (T1) to post-assessment (T3)																													
DCL-EXT Total score	TAU	-0.24	-0.29	-0.18	-0.35	-0.41	-0.29																						
	WASH	-0.34	-0.42	-0.25	-0.39	-0.49	-0.30				.006	124	56	-0.12	-0.43	0.20	.472	124	74	-0.46	-0.75	-0.17	.002	56	74	-0.34	-0.69	0.00	.053
	WASH+S	-0.41	-0.48	-0.34	-0.51	-0.59	-0.43																						
SCL-ADHD Total score	TAU	-0.17	-0.24	-0.10	-0.27	-0.35	-0.20																						
	WASH	-0.27	-0.38	-0.16	-0.36	-0.47	-0.25				.380	122	50	-0.21	-0.54	0.12	.217	122	73	-0.15	-0.44	0.14	.327	50	73	0.06	-0.30	0.42	.741
	WASH+S	-0.33	-0.42	-0.24	-0.33	-0.43	-0.24																						
SCL-DBD ODD scale	TAU	-0.06	-0.15	0.02	-0.14	-0.24	-0.04																						
	WASH	-0.17	-0.29	-0.05	-0.28	-0.43	-0.13				.072	121	50	-0.25	-0.58	0.08	.137	121	72	-0.31	-0.61	-0.02	.036	50	72	-0.07	-0.43	0.29	.719
	WASH+S	-0.20	-0.30	-0.09	-0.32	-0.45	-0.19																						
CBCL	TAU	-1.69	-2.55	-0.84	-2.08	-3.04	-1.12																						
Internalizing	WASH	-2.44	-3.74	-1.14	-1.79	-3.29	-0.30				.086	116	50	0.05	-0.28	0.39	.752	116	71	-0.29	-0.59	0.00	.054	50	71	-0.34	-0.70	0.02	.065
symptoms	WASH+S	-2.65	-3.75	-1.55	-3.64	-4.87	-2.41																						
CBCL	TAU	-2.43	-3.40	-1.46	-2.64	-3.72	-1.55																						
Externalizing	WASH	-2.76	-4.22	-1.29	-4.00	-5.69	-2.31				.236	116	50	-0.22	-0.55	0.11	.190	116	71	-0.21	-0.51	0.08	.156	50	71	0.01	-0.35	0.37	.956
symptoms	WASH+S	-2.67	-3.92	-1.41	-3.94	-5.33	-2.54																						
SCL-ADHD	TAU	-0.22	-0.31	-0.12	-0.53	-0.60	-0.47																						
Functional	WASH	-0.30	-0.44	-0.16	-0.60	-0.71	-0.50				.108	116	49	-0.19	-0.53	0.14	.260	116	71	-0.31	-0.60	-0.01	.044	49	71	-0.11	-0.47	0.25	.555
Impairment	WASH+S	-0.35	-0.46	-0.23	-0.64	-0.73	-0.56																						
KIDSCREEN Total score	TAU	0.62	-0.19	1.43	0.65	-0.24	1.55																						
	WASH	-0.21	-1.44	1.01	0.17	-1.23	1.57				.678	116	50	-0.10	-0.43	0.24	.574	116	71	0.07	-0.23	0.36	.661	50	71	0.16	-0.20	0.52	.384
	WASH+S	0.83	-0.21	1.87	0.98	-0.17	2.14																						
FPNE	TAU	-0.02	-0.06	0.02	0.01	-0.04	0.06																						
Positive	WASH	0.00	-0.06	0.06	-0.03	-0.11	0.04				.360	116	50	-0.17	-0.50	0.16	.322	116	71	0.09	-0.20	0.39	.533	50	71	0.26	-0.10	0.62	.159
parenting	WASH+S	0.04	-0.02	0.09	0.03	-0.03	0.09																						
FPNE	TAU	-0.03	-0.07	0.01	-0.07	-0.12	-0.03																						
Negative	WASH	-0.09	-0.15	-0.03	-0.10	-0.16	-0.03				.090	116	50	-0.10	-0.43	0.23	.558	116	71	-0.33	-0.62	-0.03	.032	50	71	-0.22	-0.58	0.14	.231
parenting	WASH+S	-0.14	-0.20	-0.09	-0.15	-0.21	-0.10																						
DASS Total score	TAU	-0.05	-0.11	0.01	-0.09	-0.16	-0.02																						
	WASH	-0.07	-0.15	0.02	-0.04	-0.15	0.08				.337	116	50	0.13	-0.20	0.46	.454	116	71	-0.14	-0.44	0.15	.352	50	71	-0.27	-0.63	0.10	.151
	WASH+S	-0.17	-0.24	-0.09	-0.15	-0.24	-0.05																						
Change from baseline (T1) to follow-up (T4)																													
DCL-EXT Total score	TAU	-0.24	-0.29	-0.18	-0.36	-0.42	-0.29	-0.42	-0.49	-0.35																			
	WASH	-0.34	-0.42	-0.26	-0.40	-0.49	-0.30	-0.44	-0.55	-0.34	.098	126	58	-0.07	-0.38	0.25	.681	126	74	-0.31	-0.60	-0.02	.035	58	74	-0.24	-0.58	0.11	.180
	WASH+S	-0.41	-0.48	-0.34	-0.51	-0.59	-0.43	-0.54	-0.62	-0.45																			
	TAU	-0.17	-0.24	-0.10	-0.27	-0.35	-0.20	-0.34	-0.43	-0.25	.158	125	51	-0.26	-0.59	0.06	.118	125	76	-0.23	-0.51	0.06	.117						

Variable	Condition	T2 *M*	T2 (LB;	T2 UB)	T3 *M*	T3 (LB;	T3 UB)	T4 *M*	T4 (LB;	T4 UB)
SCL-ADHD Total score	WASH	-0.27	-0.38	-0.16	-0.35	-0.46	-0.24	-0.47	-0.60	-0.34
	WASH+S	-0.32	-0.41	-0.23	-0.34	-0.43	-0.24	-0.45	-0.56	-0.35
SCL-DBD ODD scale	TAU	-0.07	-0.15	0.01	-0.14	-0.24	-0.04	-0.22	-0.32	-0.13
	WASH	-0.17	-0.29	-0.05	-0.28	-0.43	-0.13	-0.35	-0.49	-0.20
	WASH+S	-0.19	-0.30	-0.09	-0.32	-0.44	-0.19	-0.40	-0.52	-0.29
CBCL Internalizing symptoms	TAU	-1.74	-2.60	-0.88	-2.10	-3.06	-1.15	-2.76	-3.77	-1.75
	WASH	-2.27	-3.57	-0.97	-1.51	-3.00	-0.02	-3.14	-4.68	-1.59
	WASH+S	-2.62	-3.72	-1.52	-3.56	-4.79	-2.33	-3.35	-4.65	-2.06
CBCL Externalizing symptoms	TAU	-2.47	-3.44	-1.50	-2.71	-3.78	-1.63	-4.67	-5.87	-3.47
	WASH	-2.64	-4.10	-1.17	-3.82	-5.49	-2.16	-5.37	-7.21	-3.52
	WASH+S	-2.65	-3.90	-1.40	-3.95	-5.32	-2.58	-4.95	-6.50	-3.40
SCL-ADHD Functional Impairment	TAU	-0.22	-0.31	-0.13	-0.54	-0.60	-0.47	-0.60	-0.68	-0.52
	WASH	-0.30	-0.44	-0.16	-0.60	-0.70	-0.49	-0.71	-0.83	-0.59
	WASH+S	-0.34	-0.46	-0.22	-0.64	-0.72	-0.56	-0.81	-0.92	-0.71
KIDSCREEN Total score	TAU	0.60	-0.21	1.41	0.67	-0.22	1.56	0.86	-0.12	1.85
	WASH	-0.22	-1.45	1.00	0.05	-1.34	1.44	0.57	-0.88	2.02
	WASH+S	0.82	-0.23	1.86	0.77	-0.37	1.91	-0.52	-1.73	0.70
FPNE Positive parenting	TAU	-0.02	-0.06	0.02	0.01	-0.03	0.06	0.02	-0.04	0.09
	WASH	0.00	-0.06	0.06	-0.03	-0.11	0.04	-0.03	-0.13	0.06
	WASH+S	0.04	-0.02	0.09	0.03	-0.03	0.09	-0.16	-0.24	-0.08
FPNE Negative parenting	TAU	-0.03	-0.07	0.01	-0.08	-0.12	-0.03	-0.10	-0.14	-0.05
	WASH	-0.09	-0.15	-0.03	-0.09	-0.16	-0.03	-0.09	-0.16	-0.03
	WASH+S	-0.14	-0.19	-0.09	-0.14	-0.20	-0.09	-0.14	-0.19	-0.09
DASS Total score	TAU	-0.05	-0.11	0.00	-0.09	-0.16	-0.02	-0.11	-0.17	-0.05
	WASH	-0.06	-0.14	0.03	-0.02	-0.13	0.09	-0.10	-0.20	0.00
	WASH+S	-0.17	-0.24	-0.09	-0.14	-0.24	-0.05	-0.18	-0.26	-0.10

Variable	Total effect *p*	TAU vs. WASH n_{TAU}	n_{WASH}	*d*	(LB;	UB)	p_d	TAU vs. WASH+S n_{TAU}	n_{WASH+S}	*d*	(LB;	UB)	p_d	WASH vs. WASH+S n_{WASH}	n_{WASH+S}	*d*	(LB;	UB)	p_d
SCL-ADHD Total score														51	76	0.03	-0.32	0.39	.860
SCL-DBD ODD scale	.060	125	51	-0.23	-0.55	0.10	.175	125	76	-0.33	-0.62	-0.05	.023	51	76	-0.11	-0.46	0.24	.543
CBCL Internalizing symptoms	.768	119	51	-0.07	-0.39	0.26	.690	119	73	-0.10	-0.40	0.19	.482	51	73	-0.04	-0.40	0.32	.836
CBCL Externalizing symptoms	.823	119	51	-0.10	-0.43	0.22	.538	119	73	-0.04	-0.33	0.25	.780	51	73	0.06	-0.30	0.42	.737
SCL-ADHD Functional Impairment	.006	119	50	-0.24	-0.57	0.09	.155	119	73	-0.48	-0.77	-0.18	.002	50	73	-0.24	-0.60	0.12	.199
KIDSCREEN Total score	.213	119	51	-0.05	-0.38	0.27	.744	119	72	-0.26	-0.55	0.03	.086	51	72	-0.20	-0.56	0.15	.264
FPNE Positive parenting	.003	119	51	-0.15	-0.48	0.18	.364	119	73	-0.50	-0.79	-0.21	.001	51	73	-0.35	-0.71	0.00	.054
FPNE Negative parenting	.405	119	51	0.02	-0.31	0.34	.926	119	73	-0.18	-0.47	0.11	.224	51	73	-0.20	-0.56	0.16	.279
DASS Total score	.312	119	51	0.02	-0.30	0.35	.884	119	73	-0.20	-0.50	0.09	.171	51	73	-0.23	-0.59	0.13	.211

Note. TAU = treatment as usual, WASH = web-assisted self-help, WASH+S= web-assisted self-help and additional support via telephone, DCL-EXT = Diagnostic Checklist for Externalizing Behavior Disorders, SCL-ADHD = Symptom Checklist for Attention-Deficit/Hyperactivity Disorder, SCL-DBD = Symptom Checklist for Disruptive Behavior Disorders, ODD = Oppositional Defiant Disorder, CBCL = Child Behavior Checklist, KIDSCREEN-10 = questionnaire to assess health-related quality of life, FPNE = Assessment Scale of Positive and Negative Parenting Behavior, DASS = Depression Anxiety Stress Scales, T1 = baseline, T2 = interim assessment (3 months post-baseline), T3 = post-assessment (6 months post-baseline) T4 = follow-up (6 months post-T3). *M* = mean, LB = lower bound of 95% confidence interval, UB = upper bound of 95% confidence interval, *p* = significance value, *n* = sample size, *d* = Cohen's *d* (effect size).

Table S9

Group comparisons regarding the mean change from baseline (T_1) to post-assessment (T_3) and from baseline to follow-up (T_4) based on mixed model repeated measures (MMRM) analyses: per-protocol sample (WASH: made use of at least 40% of the WASH intervention; WASH+S: additionally participated in at least 3 telephone consultations)

		Marginal means									Total effect	Pairwise comparisons																	
		T2			T3			T4				TAU vs. WASH						TAU vs. WASH+S						WASH vs. WASH+S					
Variable	Condition	*M*	*(LB;*	*UB)*	*M*	*(LB;*	*UB)*	*M*	*(LB;*	*UB)*	*p*	n_{TAU}	n_{WASH}	*d*	*(LB;*	*UB)*	p_d	n_{TAU}	n_{WASH+S}	*d*	*(LB;*	*UB)*	p_d	n_{WASH}	n_{WASH+S}	*d*	*(LB;*	*UB)*	p_d
Change from baseline (T1) to post-assessment (T3)																													
DCL-EXT	TAU	-0.23	-0.29	-0.18	-0.35	-0.41	-0.29																						
Total score	WASH	-0.32	-0.41	-0.23	-0.38	-0.48	-0.27				.002	124	44	-0.08	-0.42	0.26	.651	124	62	-0.53	-0.83	-0.22	.001	44	62	-0.45	-0.83	-0.06	.024
	WASH+S	-0.41	-0.48	-0.33	-0.53	-0.62	-0.45																						
SCL-ADHD	TAU	-0.16	-0.23	-0.09	-0.27	-0.34	-0.20																						
Total score	WASH	-0.28	-0.40	-0.16	-0.33	-0.45	-0.20				.648	122	40	-0.14	-0.49	0.22	.451	122	60	-0.11	-0.42	0.19	.467	40	60	0.02	-0.38	0.42	.910
	WASH+S	-0.33	-0.43	-0.23	-0.32	-0.42	-0.22																						
SCL-DBD	TAU	-0.06	-0.14	0.02	-0.13	-0.23	-0.03																						
ODD scale	WASH	-0.14	-0.28	0.01	-0.29	-0.46	-0.13				.083	121	40	-0.29	-0.65	0.06	.110	121	59	-0.31	-0.62	0.01	.056	40	59	-0.01	-0.41	0.39	.958
	WASH+S	-0.16	-0.28	-0.05	-0.30	-0.44	-0.16																						
CBCL	TAU	-1.69	-2.56	-0.83	-2.07	-3.01	-1.13																						
Internalizing	WASH	-2.87	-4.34	-1.40	-1.77	-3.41	-0.13				.102	116	40	0.06	-0.30	0.42	.758	116	59	-0.30	-0.62	0.01	.058	40	59	-0.36	-0.76	0.05	.084
symptoms	WASH+S	-3.02	-4.24	-1.80	-3.65	-4.95	-2.35																						
CBCL	TAU	-2.38	-3.33	-1.43	-2.54	-3.58	-1.50																						
Externalizing	WASH	-3.22	-4.84	-1.61	-4.07	-5.90	-2.24				.207	116	40	-0.26	-0.62	0.10	.160	116	59	-0.22	-0.54	0.09	.162	40	59	0.04	-0.36	0.44	.847
symptoms	WASH+S	-2.40	-3.74	-1.05	-3.84	-5.29	-2.39																						
SCL-ADHD	TAU	-0.21	-0.31	-0.12	-0.53	-0.60	-0.46																						
Functional	WASH	-0.34	-0.50	-0.18	-0.61	-0.73	-0.49				.188	116	39	-0.21	-0.57	0.15	.258	116	59	-0.27	-0.58	0.05	.095	39	59	-0.05	-0.46	0.35	.803
Impairment	WASH+S	-0.32	-0.45	-0.19	-0.63	-0.72	-0.54																						
KIDSCREEN	TAU	0.52	-0.28	1.33	0.56	-0.32	1.43																						
Total score	WASH	-0.58	-1.94	0.78	0.30	-1.24	1.85				.855	116	40	-0.05	-0.41	0.31	.783	116	59	0.06	-0.25	0.37	.706	40	59	0.11	-0.29	0.51	.590
	WASH+S	0.86	-0.28	2.00	0.85	-0.37	2.07																						
FPNE	TAU	-0.02	-0.06	0.02	0.01	-0.04	0.06																						
Positive	WASH	0.02	-0.05	0.09	-0.02	-0.10	0.06				.599	116	40	-0.12	-0.48	0.24	.518	116	59	0.09	-0.23	0.40	.588	40	59	0.20	-0.20	0.61	.318
parenting	WASH+S	0.03	-0.03	0.08	0.03	-0.03	0.10																						
FPNE	TAU	-0.03	-0.07	0.01	-0.07	-0.11	-0.03																						
Negative	WASH	-0.08	-0.15	-0.01	-0.08	-0.15	0.00				.022	116	40	-0.01	-0.37	0.35	.939	116	59	-0.42	-0.73	-0.11	.009	40	59	-0.40	-0.80	0.01	.055
parenting	WASH+S	-0.15	-0.21	-0.10	-0.17	-0.23	-0.11																						
DASS Total	TAU	-0.05	-0.11	0.01	-0.09	-0.16	-0.02																						
score	WASH	-0.06	-0.16	0.04	-0.06	-0.18	0.07				.289	116	40	0.08	-0.28	0.44	.670	116	59	-0.21	-0.52	0.10	.193	40	59	-0.28	-0.69	0.12	.168
	WASH+S	-0.15	-0.24	-0.07	-0.17	-0.26	-0.07																						
Change from baseline (T1) to follow-up (T4)																													
	TAU	-0.24	-0.29	-0.18	-0.35	-0.42	-0.29	-0.42	-0.48	-0.35																			
DCL-EXT	WASH	-0.32	-0.41	-0.23	-0.38	-0.49	-0.28	-0.42	-0.53	-0.31	.035	126	46	-0.02	-0.36	0.32	.912	126	62	-0.39	-0.69	-0.08	.014*	46	62	-0.36	-0.74	0.02	.067
Total score	WASH+S	-0.41	-0.48	-0.33	-0.53	-0.62	-0.45	-0.55	-0.64	-0.47																			

Variable	Condition	Marginal means T2 *M*	T2 (*LB*;	T2 *UB*)	T3 *M*	T3 (*LB*;	T3 *UB*)	T4 *M*	T4 (*LB*;	T4 *UB*)	Total effect *p*	TAU vs. WASH n_{TAU}	n_{WASH}	*d*	(*LB*;	*UB*)	p_d	TAU vs. WASH+S n_{TAU}	n_{WASH+S}	*d*	(*LB*;	*UB*)	p_d	WASH vs. WASH+S n_{WASH}	n_{WASH+S}	*d*	(*LB*;	*UB*)	p_d
SCL-ADHD Total score	TAU	-0.17	-0.24	-0.10	-0.27	-0.34	-0.20	-0.34	-0.42	-0.25																			
	WASH	-0.28	-0.40	-0.16	-0.32	-0.44	-0.19	-0.44	-0.58	-0.30	.160	125	41	-0.22	-0.58	0.13	.214	125	62	-0.27	-0.58	0.03	.081	41	62	-0.05	-0.44	0.35	.809
	WASH+S	-0.32	-0.42	-0.22	-0.33	-0.43	-0.23	-0.46	-0.58	-0.35																			
SCL-DBD ODD scale	TAU	-0.06	-0.14	0.02	-0.13	-0.23	-0.03	-0.22	-0.31	-0.12																			
	WASH	-0.14	-0.28	0.01	-0.29	-0.46	-0.13	-0.31	-0.47	-0.15	.125	125	41	-0.18	-0.53	0.18	.332	125	62	-0.31	-0.61	-0.01	.048	41	62	-0.14	-0.53	0.26	.491
	WASH+S	-0.16	-0.28	-0.04	-0.31	-0.44	-0.17	-0.38	-0.52	-0.25																			
CBCL Internalizing symptoms	TAU	-1.73	-2.60	-0.86	-2.08	-3.02	-1.14	-2.73	-3.74	-1.71																			
	WASH	-2.67	-4.14	-1.19	-1.46	-3.10	0.17	-2.94	-4.68	-1.21	.591	119	41	-0.04	-0.39	0.32	.832	119	61	-0.16	-0.47	0.15	.311	41	61	-0.12	-0.52	0.27	.547
	WASH+S	-2.96	-4.18	-1.74	-3.62	-4.92	-2.32	-3.64	-5.07	-2.21																			
CBCL Externalizing symptoms	TAU	-2.43	-3.38	-1.48	-2.61	-3.64	-1.58	-4.60	-5.80	-3.40																			
	WASH	-3.08	-4.70	-1.47	-3.91	-5.71	-2.11	-5.37	-7.43	-3.31	.803	119	41	-0.12	-0.47	0.24	.526	119	61	0.00	-0.31	0.31	.991	41	61	0.11	-0.28	0.51	.579
	WASH+S	-2.38	-3.73	-1.04	-3.87	-5.30	-2.43	-4.61	-6.32	-2.90																			
SCL-ADHD Functional Impairment	TAU	-0.22	-0.32	-0.13	-0.54	-0.60	-0.47	-0.60	-0.68	-0.52																			
	WASH	-0.34	-0.50	-0.18	-0.60	-0.72	-0.48	-0.74	-0.87	-0.61	.006	119	40	-0.32	-0.68	0.04	.082	119	61	-0.49	-0.80	-0.18	.00	40	61	-0.17	-0.57	0.23	.402
	WASH+S	-0.32	-0.45	-0.18	-0.63	-0.72	-0.53	-0.81	-0.92	-0.71																			
KIDSCREEN Total score	TAU	0.52	-0.28	1.33	0.59	-0.29	1.46	0.79	-0.20	1.78																			
	WASH	-0.57	-1.93	0.79	0.19	-1.34	1.73	0.16	-1.48	1.79	.289	119	41	-0.12	-0.47	0.24	.520	119	60	-0.25	-0.56	0.06	.120	41	60	-0.13	-0.53	0.27	.516
	WASH+S	0.84	-0.31	1.98	0.69	-0.52	1.90	-0.55	-1.90	0.80																			
FPNE Positive parenting	TAU	-0.02	-0.06	0.02	0.01	-0.03	0.06	0.02	-0.04	0.09																			
	WASH	0.02	-0.05	0.09	-0.02	-0.10	0.06	-0.06	-0.16	0.05	.001	119	41	-0.21	-0.57	0.14	.238	119	61	-0.60	-0.91	-0.29	≤.001	41	61	-0.39	-0.78	0.01	.057
	WASH+S	0.03	-0.03	0.08	0.03	-0.04	0.09	-0.19	-0.28	-0.10																			
FPNE Negative parenting	TAU	-0.03	-0.07	0.01	-0.08	-0.12	-0.03	-0.10	-0.14	-0.05																			
	WASH	-0.08	-0.15	-0.01	-0.07	-0.14	0.00	-0.08	-0.15	-0.01	.224	119	41	0.07	-0.28	0.43	.681	119	61	-0.23	-0.54	0.08	.144	41	61	-0.31	-0.70	0.09	.129
	WASH+S	-0.15	-0.21	-0.09	-0.16	-0.22	-0.11	-0.15	-0.21	-0.09																			
DASS Total score	TAU	-0.05	-0.11	0.01	-0.09	-0.15	-0.02	-0.11	-0.17	-0.04																			
	WASH	-0.05	-0.15	0.04	-0.04	-0.16	0.08	-0.09	-0.20	0.02	.382	119	41	0.04	-0.31	0.40	.822	119	61	-0.19	-0.50	0.11	.218	41	61	-0.24	-0.63	0.16	.244
	WASH+S	-0.15	-0.23	-0.07	-0.16	-0.26	-0.07	-0.18	-0.27	-0.09																			

Note. TAU = treatment as usual, WASH = web-assisted self-help, WASH+S= web-assisted self-help and additional support via telephone, DCL-EXT = Diagnostic Checklist for Externalizing Behavior Disorders, SCL-ADHD = Symptom Checklist for Attention-Deficit/Hyperactivity Disorder, SCL-DBD = Symptom Checklist for Disruptive Behavior Disorders, ODD = Oppositional Defiant Disorder, CBCL = Child Behavior Checklist, KIDSCREEN = questionnaire to assess health-related quality of life, FPNE = Assessment Scale of Positive and Negative Parenting Behavior, DASS = Depression Anxiety Stress Scales, T1 = baseline, T2 = interim assessment, T3 = post-assessment, T4 = follow-up. *M* = mean, LB = lower bound of 95% confidence interval, UB = upper bound of 95% confidence interval, *p* = significance value, *n* = sample size, *d* = Cohen's *d* (effect size).

Table S10

Group comparisons regarding the mean DCL-EXT change from baseline (T_1) to post-assessment (T_3) based on mixed model repeated measures (MMRM) analyses: sensitivity analyses in the intention-to-treat sample

		Marginal means						Total effect	Pairwise comparisons																	
		T2			T3				TAU vs. WASH						TAU vs. WASH+S						WASH vs. WASH+S					
Variable	Condition	*M*	(LB;	UB)	*M*	(LB;	UB)	*p*	n_{TAU}	n_{WASH}	*d*	(LB;	UB)	p_d	n_{TAU}	n_{WASH+S}	*d*	(LB;	UB)	p_d	n_{WASH}	n_{WASH+S}	*d*	(LB;	UB)	p_d
	TAU	-0.23	-0.29	-0.18	-0.35	-0.41	-0.28																			
All cases	WASH	-0.29	-0.35	-0.23	-0.36	-0.43	-0.29	.018	123	114	-0.02	-0.27	0.24	.886	123	121	-0.33	-0.58	-0.08	.010	121	114	-0.30	-0.56	-0.05	.020
	WASH+S	-0.38	-0.44	-0.32	-0.47	-0.54	-0.41																			
Cases with	TAU	-0.24	-0.31	-0.16	-0.36	-0.46	-0.27																			
formal	WASH	-0.32	-0.40	-0.25	-0.40	-0.49	-0.31	.232	72	76	-0.09	-0.42	0.12	.571	72	73	-0.28	-0.60	0.05	.098	76	73	-0.18	-0.50	0.14	.268
diagnosis	WASH+S	-0.37	-0.45	-0.29	-0.48	-0.57	-0.39																			
Cases without	TAU	-0.23	-0.32	-0.15	-0.32	-0.42	-0.23																			
formal	WASH	-0.25	-0.35	-0.15	-0.31	-0.42	-0.19	.060	51	38	0.04	-0.38	0.46	.835	51	48	-0.40	-0.79	-0.01	.049	38	48	-0.43	-0.86	-0.01	.048
diagnosis	WASH+S	-0.38	-0.47	-0.29	-0.46	-0.56	-0.36																			

Note. All analyses controlled for the use of non-study pharmacological and non-pharmacological interventions. The analysis in all cases included diagnostic status (cases with formal diagnosis vs. cases without formal diagnosis) as additional predictor. TAU = treatment as usual, WASH = web-assisted self-help, WASH+S= web-assisted self-help and additional support via telephone, T2 = interim assessment (3 months post-baseline), T3 = post-assessment (6 months post-baseline). *M* = mean, LB = lower bound of 95% confidence interval, UB = upper bound of 95% confidence interval, *p* = significance value, *n* = sample size, *d* = Cohen's *d* (effect size).

Table S11

Prediction of blinded clinician-rated child externalizing symptoms (primary outcome) at T3 in the TAU condition: intention-to-treat sample

	β	*SE*	*t*	p_t	$CI_{95\%}$	
Complete model with all putative predictors[1)]						
Blinded clinician-rated child externalizing symptoms at baseline (DCL-EXT total)	0.750	0.108	6.95	<.001	0.536	0.965
Age of participating caregiver at baseline (years)	-0.004	0.007	-0.62	.536	-0.019	0.010
Sex of participating caregiver - female	-0.048	0.124	-0.38	.703	-0.295	0.200
Speciality of the referring physician – child and adolescent psychiatrist	0.030	0.073	0.41	.685	-0.115	0.174
Supply area [urban vs. rural] – rural	-0.004	0.095	-0.04	.968	-0.194	0.186
Educational level of participating caregiver				.924[3)]		
• Upper secondary level of education (ISCED level 3B)[a]	0.092	0.117	0.79	(.432)	-0.140	0.325
• Upper secondary level of education (ISCED level 3A) and post-secondary non-tertiary (ISCED level 4)[b]	0.098	0.134	0.73	(.465)	-0.168	0.365
• First stage of tertiary education (ISCED level 5B)[c]	0.106	0.125	0.85	(.400)	-0.143	0.354
• First stage (ISCED level 5A) or second stage of tertiary education (ISCED level 6)[d]	0.110	0.126	0.87	(.385)	-0.140	0.361
Caregiver currently employed - yes	-0.010	0.097	-0.11	.916	-0.203	0.183
Positive parenting behavior (FPNE positive parenting) at baseline	-0.106	0.107	-0.99	.325	-0.320	0.107
Negative parenting behavior (FPNE negative parenting at baseline	-0.081	0.151	-0.54	.594	-0.381	0.220
Parental internalizing symptoms (DASS total score) at baseline	-0.067	0.099	-0.67	.503	-0.263	0.130
Child age (years)	-0.009	0.022	-0.42	.672	-0.052	0.034
Child sex -female	-0.010	0.088	-0.12	.906	-0.185	0.164
Child attending special school - yes	0.189	0.153	1.24	.219	-0.114	0.493
Child quality of life (KIDSCREEN total score) at baseline	0.009	0.009	1.04	.299	-0.008	0.026
Functional impairment (SCL-ADHD Functional Impairment) at baseline	0.063	0.073	0.86	.390	-0.082	0.208
Functional impairment (SCL-DBD Functional Impairment) at baseline	-0.084	0.074	-1.13	.261	-0.231	0.064
Behavioral and emotional problems (CBCL total) at baseline	0.005	0.002	2.27	.025	0.001	0.009
Intercept	0.153	0.684	0.22	.823	-1.208	1.515
Final model with remaining predictors[2)]						
Blinded clinician-rated child externalizing symptoms at baseline (DCL-EXT total)	0.710	0.093	7.62	<.001	0.526	0.895
Child attending special school - yes	0.188	0.142	1.33	.187	-0.092	0.468
Behavioral and emotional problems (CBCL total) at baseline	0.003	0.002	2.08	.040	0.000	0.006
Intercept	-0.119	0.128	-0.93	.356	-0.372	0.135

Notes. [1)]$F_{(20.\ 117.8)}$= 5.39. $p \leq .001$. R^2=0.47; [2)]$F_{(3.133.2)}$=38.81. $p \leq .001$. R^2=0.49; [3)]Joint test that all coefficients of the dummy coded categories are equal to 0 (over-all criterion).

[a] basic vocational training year, vocational schools, specialized vocational schools, commercial school

[b] vocational high school, specialized vocational high school, specialized vocational schools: qualification for ISCED 5A, "Kollegschulen": qualification for ISCED 5A, upper secondary schools (German diplomas: Fachhochschulreife, Hochschulreife)

[c] trade and technical school (German diplomas: Meister/Techniker, Fachschulabschluss), health sector school, vocational academy

[d] university, German "Fachhochschule", doctoral studies (diplomas: Diplom (FH). university diploma, Bachelor, Master, PhD)

Table S12

Prediction of blinded clinician-rated child externalizing symptoms (primary outcome) at T3 in the WASH+TAU condition: intention-to-treat sample

	β	*SE*	*t*	p_t	$KI_{95\%}$	
Complete model with all putative predictors[1)]						
Blinded clinician-rated child externalizing symptoms at baseline (DCL-EXT total)	0.581	0.115	5.04	<.001	0.352	0.810
Age of participating caregiver at baseline (years)	-0.006	0.007	-0.83	.408	-0.020	0.008
Sex of participating caregiver - female	-0.117	0.132	-0.89	.379	-0.379	0.145
Speciality of the referring physician – child and adolescent psychiatrist	-0.069	0.087	-0.79	.433	-0.244	0.106
Supply area [urban vs. rural] – rural	0.015	0.102	0.15	.882	-0.187	0.217
Educational level of participating caregiver				.097[3)]		
• Upper secondary level of education (ISCED level 3B)[a]	0.323	0.124	2.59	(.011)	0.075	0.570
• Upper secondary level of education (ISCED level 3A) and post-secondary non-tertiary (ISCED level 4)[b]	0.271	0.140	1.93	(.057)	-0.008	0.550
• First stage of tertiary education (ISCED level 5B)[c]	0.252	0.156	1.62	(.110)	-0.058	0.562
• First stage (ISCED level 5A) or second stage of tertiary education (ISCED level 6)[d]	0.335	0.123	2.72	(.008)	0.091	0.580
Caregiver currently employed - yes	-0.196	0.103	-1.91	0.059	-0.401	0.008
Positive parenting behavior (FPNE positive parenting) at baseline	0.082	0.135	0.61	0.547	-0.187	0.351
Negative parenting behavior (FPNE negative parenting at baseline	0.172	0.134	1.28	0.205	-0.095	0.439
Parental internalizing symptoms (DASS total score) at baseline	0.092	0.106	0.88	0.384	-0.117	0.302
Child age (years)	-0.014	0.023	-0.60	0.551	-0.061	0.033
Child sex -female	-0.112	0.099	-1.14	0.260	-0.308	0.084
Child attending special school - yes	0.021	0.181	0.11	0.909	-0.339	0.380
Child quality of life (KIDSCREEN total score) at baseline	0.000	0.009	0.00	1.000	-0.018	0.018
Functional impairment (SCL-ADHD Functional Impairment) at baseline	-0.013	0.087	-0.15	0.882	-0.186	0.160
Functional impairment (SCL-DBD Functional Impairment) at baseline	0.001	0.074	0.01	0.992	-0.146	0.147
Behavioral and emotional problems (CBCL total) at baseline	0.000	0.002	-0.07	0.944	-0.005	0.004
At least one login - yes	-0.048	0.122	-0.40	0.693	-0.292	0.195
Total time of use of the WASH program (hours)	-0.004	0.008	-0.47	0.643	-0.021	0.013
Percentage of tasks processed	-0.001	0.002	-0.42	0.674	-0.004	0.003
Intercept	0.169	0.770	0.22	0.827	-1.364	1.702
Final model with remaining predictors[2)]						
Blinded clinician-rated child externalizing symptoms at baseline (DCL-EXT total)	0.639	0.095	6.76	<.001	0.452	0.827
Educational level of participating caregiver				.129[3)]		
• Upper secondary level of education (ISCED level 3B)[a]	0.295	0.116	2.55	(.012)	0.065	0.525
• Upper secondary level of education (ISCED level 3A) and post-secondary non-tertiary (ISCED level 4)[b]	0.225	0.132	1.71	(.092)	-0.037	0.488
• First stage of tertiary education (ISCED level 5B)[c]	0.241	0.145	1.66	(.100)	-0.047	0.529
• First stage (ISCED level 5A) or second stage of tertiary education (ISCED level 6)[d]	0.274	0.113	2.43	(.017)	0.050	0.499
Caregiver currently employed - yes	-0.175	0.094	-1.87	.064	-0.360	0.011
Negative parenting behavior (FPNE negative parenting at baseline	0.175	0.116	1.51	.136	-0.056	0.406
Intercept	-0.239	0.304	-0.78	0.435	-0.843	0.365

Notes. [1)] $F_{(23.\ 107.3)}$= 2.75. p≤.001; R^2=0.32; [2)] $F_{(7.121.7)}$=8.45. *p*≤.001. R^2=0.34; [3)]Joint test that all coefficients of the dummy coded categories are equal to 0 (over-all criterion).

[a] basic vocational training year, vocational schools, specialized vocational schools, commercial school

[b] vocational high school, specialized vocational high school, specialized vocational schools: qualification for ISCED 5A, “Kollegschulen”: qualification for ISCED 5A, upper secondary schools (German diplomas: Fachhochschulreife, Hochschulreife)

[c] trade and technical school (German diplomas: Meister/Techniker, Fachschulabschluss), health sector school, vocational academy

[d] university, German "Fachhochschule", doctoral studies (diplomas: Diplom (FH). university diploma, Bachelor, Master, PhD)

Table S13

Prediction of blinded clinician-rated child externalizing symptoms (primary outcome) at T3 in the WASH+S+TAU condition: intention-to-treat sample

	β	*SE*	*t*	p_t	$KI_{95\%}$	
Complete model with all putative predictors[1)]						
Blinded clinician-rated child externalizing symptoms at baseline (DCL-EXT total)	0.631	0.109	5.77	<.001	0.414	0.848
Age of participating caregiver at baseline (years)	-0.005	0.006	-0.78	.440	-0.017	0.008
Sex of participating caregiver - female	-0.007	0.127	-0.06	.955	-0.259	0.244
Specialty of the referring physician – child and adolescent psychiatrist	0.125	0.071	1.75	.083	-0.017	0.266
Supply area [urban vs. rural] – rural	-0.012	0.095	-0.13	.896	-0.201	0.176
Educational level of participating caregiver				.510[3)]		
• Upper secondary level of education (ISCED level 3B)[a]	-0.148	0.119	-1.25	(.215)	-0.384	0.088
• Upper secondary level of education (ISCED level 3A) and post-secondary non-tertiary (ISCED level 4)[b]	-0.022	0.111	-0.20	(.842)	-0.244	0.199
• First stage of tertiary education (ISCED level 5B)[c]	0.049	0.153	0.32	(.749)	-0.256	0.354
• First stage (ISCED level 5A) or second stage of tertiary education (ISCED level 6)[d]	-0.002	0.116	-0.01	(.990)	-0.232	0.229
Caregiver currently employed - yes	-0.130	0.102	-1.28	.204	-0.333	0.072
Positive parenting behavior (FPNE positive parenting) at baseline	-0.180	0.110	-1.64	.105	-0.398	0.038
Negative parenting behavior (FPNE negative parenting at baseline	-0.007	0.136	-0.05	.957	-0.278	0.263
Parental internalizing symptoms (DASS total score) at baseline	0.007	0.091	0.07	.941	-0.175	0.189
Child age (years)	0.056	0.022	2.50	.014	0.012	0.100
Child sex -female	-0.041	0.095	-0.44	.663	-0.230	0.147
Child attending special school - yes	0.043	0.144	0.30	.768	-0.243	0.328
Child quality of life (KIDSCREEN total score) at baseline	0.001	0.008	0.12	.904	-0.015	0.017
Functional impairment (SCL-ADHD Functional Impairment) at baseline	0.010	0.068	0.15	.878	-0.125	0.146
Functional impairment (SCL-DBD Functional Impairment) at baseline	-0.065	0.073	-0.88	.382	-0.210	0.081
Behavioral and emotional problems (CBCL total) at baseline	0.004	0.002	1.81	.074	-0.000	0.008
At least one login - yes	0.312	0.138	2.26	.027	0.037	0.588
Total time of use of the WASH program (hours)	0.005	0.004	1.41	.162	-0.002	0.013
Percentage of tasks processed	-0.004	0.002	-2.61	.011	-0.007	-0.001
Number of support calls	-0.011	0.020	-0.54	.593	-0.051	0.029
Intercept	0.140	0.682	0.20	.838	-1.216	1.495
Finales Modell mit verbliebenen Prädiktoren[2)]						
Blinded clinician-rated child externalizing symptoms at baseline (DCL-EXT total)	0.648	0.099	6.58	<.001	0.453	0.844
Specialty of the referring physician – child and adolescent psychiatrist	0.116	0.065	1.78	.078	-0.013	0.245
Child age (years)	0.039	0.019	2.02	.047	0.001	0.078
Behavioral and emotional problems (CBCL total) at baseline	0.003	0.002	1.48	.141	-0.001	0.006
At least one login - yes	0.233	0.130	1.79	.079	-0.027	0.493
Percentage of tasks processed	-0.003	0.001	-2.30	.023	-0.006	-0.000
Intercept	-0.612	0.271	-2.26	.027	-1.152	-0.073

Notes. [1)]$F_{(24.\ 111.5)}$= 4.43. p≤.001. R^2=0.45; [2)]$F_{(6.126.7)}$=14.98. p≤.001. R^2=0.44; [3)]Joint test that all coefficients of the dummy coded categories are equal to 0 (over-all criterion).

[a] basic vocational training year, vocational schools, specialized vocational schools, commercial school

[b] vocational high school, specialized vocational high school, specialized vocational schools: qualification for ISCED 5A, "Kollegschulen": qualification for ISCED 5A, upper secondary schools (German diplomas: Fachhochschulreife, Hochschulreife)

[c] trade and technical school (German diplomas: Meister/Techniker, Fachschulabschluss), health sector school, vocational academy

[d] university, German “Fachhochschule”, doctoral studies (diplomas: Diplom (FH). university diploma, Bachelor, Master, PhD)

References

Achenbach, T. M., & Rescorla, L. A. (2001). *Manual for the ASEBA school-age forms and profiles*. Burlington, VT: University of Vermont, Research Center for Children, Youth and Families.

Boß, L., Lehr, D., Reis, D., Vis, C., Riper, H., Berking, M., & Ebert, D. D. (2016). Reliability and validity of assessing user satisfaction with web-based health interventions. Journal of Medical Internet Research, 18, e234. https://doi.org/10.2196/jmir.5952

Döpfner & Görtz-Dorten (2017). *Diagnostik-System für Psychische Störungen nach ICD-10 und DSM-5 für Kinder und Jugendliche – III [Diagnostic System for Mental Disorders in Childhood and Adolescence According to ICD-10 and DSM-5]*. Göttingen: Hogrefe.

Döpfner, M., Plück, J., Kinnen, C., & Arbeitsgruppe Deutsche Child Behavior Checklist (2014). *Elternfragebogen über das Verhalten von Kindern und Jugendlichen (CBCL/6-18R). Deutschsprachige Fassung der Child Behavior Checklist for Ages 6–18 von Thomas M. Achenbach [German version of the Child Behavior Checklist for Ages 6–18 by Thomas M. Achenbach]*. Göttingen, Germany: Hogrefe.

Erhart, M., Doepfner, M., Ravens-Sieberer, U., & BELLA Study Group. (2008). Psychometric properties of two ADHD questionnaires: Comparing the Conners' scale and the FBB-HKS in the general population of German children and adolescents – results of the BELLA study. *European Child and Adolescent Psychiatry, 17*(Suppl. 1), 106–115.

Goertz-Dorten, A., Ise, E., Hautmann, C., Walter, D., & Doepfner, M. (2014). Psychometric properties of a German parent rating scale for oppositional defiant and conduct disorder (FBB-SSV) in clinical and community samples. *Child Psychiatry and Human Development*, *45*, 388–397.

Holas, V., Thöne, A. K., Dose, C., Gebauer, S., Hautmann, C., Görtz-Dorten, A., ... & Döpfner, M. (2024). Psychometric properties of the parent-rated assessment scale of positive and negative parenting behavior (FPNE) in a German sample of school-aged children. *Child and Adolescent Psychiatry and Mental Health*, *18*, 1-15.

Imort, S., Hautmann, C., Greimel, L., Katzmann, J., Pinior, J., Scholz, K., … & Doepfner, M. (2014). Fragebogen zum positiven und negativen Erziehungsverhalten [Positive and negative parenting questionnaire]. Poster presented at the 32nd Conference for Clinical Psychology and Psychotherapy of the German Society for Psychology, Braunschweig, Germany.

Ivanova, M. Y., Achenbach, T. M., Dumenci, L., Rescorla, L. A., Almqvist, F., Weintraub, S., ... & Verhulst, F. C. (2007). Testing the 8-syndrome structure of the Child Behavior Checklist in 30 societies. *Journal of Clinical Child and Adolescent Psychology*, *36*(3), 405–441.

KIDSCREEN Group Europe (2006). *The KIDSCREEN questionnaires: quality of life questionnaires for children and adolescents*. Lengerich, Germany: Pabst.

Lovibond, P. F., & Lovibond, S. H. (1995). The structure of negative emotional states: comparison of the Depression Anxiety Stress Scales (DASS) with the Beck Depression and Anxiety Inventories. *Behaviour Research and Therapy*, *33*, 335–343. https://doi.org/10.1016/0005-7967(94)00075-U

Lovibond, S. H., & Lovibond, P. F. (1995). *Manual for the Depression Anxiety Stress Scales (DASS)* (2nd ed.). *Psychology Foundation monograph.* Sydney: Psychology Foundation of Australia.

Nilges, P., & Essau, C. (2015). Die Depressions-Angst-Stress-Skalen: Der DASS--ein Screeningverfahren nicht nur für Schmerzpatienten [Depression, anxiety and stress

scales: DASS--A screening procedure not only for pain patients]. *Schmerz (Berlin, Germany)*, *29*, 649–657. https://doi.org/10.1007/s00482-015-0019-z

Organization for Economic Co-operation and Development (OECD) (1999). *Classifying educational programmes: Manual for ISCED-97 implementation in OECD countries*. Paris, France: Author.

Ravens-Sieberer, U., M. Erhart, L. Rajmil, M. Herdman, P. Auquier, J. Bruil, M. Power, W. Duer, T. Abel, L. Czemy, J. Mazur, A. Czimbalmos, Y. Tountas, C. Hagquist, J. Kilroe and European KIDSCREEN (2010). Reliability, construct and criterion validity of the KIDSCREEN-10 score: a short measure for children and adolescents' well-being and health-related quality of life. *Quality of Life Research*, *19*, 1487–1500.

Thöne, A.-K., Görtz-Dorten, A., Altenberger, P., Dose, C., Geldermann, N., Hautmann, C., Jendreizik, L. T., Treier, A.-K., von Wirth, E., Banaschewski, T., Brandeis, D., Millenet, S., Hohmann, S., Becker, K., Ketter, J., Hebebrand, J., Wenning, J., Holtmann, M., Legenbauer, T., Huss, M., Romanos, M., Jans, T., Geissler, J., Poustka, L., Uebel-von Sandersleben, H., Renner, T., Dürrwächter, U., & Döpfner, M. (2020). Toward a dimensional assessment of externalizing disorders in children: reliability and validity of a semi-structured parent interview. *Frontiers in Psychology*, *11*, 1840.
